# Experimental and DFT Investigation of a Vitamin B_6_-Derived Fluorescent Probe for Detection of Al^3+^ and Ga^3+^ Ions in a Buffered Aqueous DMSO Solution

**DOI:** 10.3390/s26092816

**Published:** 2026-04-30

**Authors:** Maksim N. Zavalishin, Artemiy A. Guschin, George A. Gamov

**Affiliations:** General Chemical Technology Department, Ivanovo State University of Chemistry and Technology, Sheremetevskii Ave. 7, 153000 Ivanovo, Russia; artemiygushiin@gmail.com (A.A.G.); ggamov@isuct.ru (G.A.G.)

**Keywords:** vitamin B_6_, hydrazone, fluorescent probe, gallium ion, aluminum ion, DFT

## Abstract

A new selective fluorescent probe based on a vitamin B_6_ derived hydrazone was synthesized and characterized for the detection of Al^3+^ and Ga^3+^ ions. The probe’s selectivity and sensitivity were evaluated using UV-Vis, fluorescence, and NMR spectroscopy in a buffered DMSO/water solution, complemented by density functional theory (DFT) calculations to elucidate the electronic structure and coordination modes of the resulting complexes. The probe exhibited a notable “turn-on” fluorescence response upon binding Al^3+^ and Ga^3+^, with emission maxima at 466 nm and 477 nm, respectively, and detection limits as low as 48 nM for Al^3+^ and 33 nM for Ga^3+^. The probe showed high selectivity for these ions over a wide range of competing cations and anions, forming stable 1:1 complexes with log *β′* values of 5.98 for Al^3+^ and 6.28 for Ga^3+^. DFT calculations revealed a tridentate coordination mode via the phenolic oxygen, azomethine nitrogen, and carbonyl oxygen, with distinct electronic transitions for each complex, including a ligand-to-metal charge transfer character in the Ga^3+^ complex. The probe demonstrates reversibility and excellent solution stability, offering a simple and sensitive platform for the environmental and biological monitoring of aluminum(III) and gallium(III) ions.

## 1. Introduction

Metal ions play a fundamental role in biological, environmental, and industrial processes. Among them, aluminum (Al^3+^), the third most common element in the Earth’s crust, has no known biological function and exhibits pronounced neurotoxicity when accumulated in the body, which is associated with the development of diseases such as Alzheimer’s disease and encephalopathy [1,2]. Its widespread use leads to increased concentrations in drinking water and soil [3]. Gallium (Ga^3+^), which has an ionic radius and chemical properties similar to those of aluminum, is widely used in the electronics industry [4,5], high-temperature thermometers [6], pharmacology (in gallium-based drugs for the treatment of hypercalcemia and certain cancers) [7], and as a diagnostic radiopharmaceutical (^67^Ga) [8,9]. Gallium(III) ions mimic iron(III) in biosystems and can also be used to fight bacteria [10,11]. However, high concentrations of gallium can also be toxic. In this regard, developing highly sensitive and selective methods for monitoring these ions in the environment and biological systems is an urgent task.

Traditional methods such as atomic absorption spectroscopy (AAS) [12], electrochemistry [13], chromatography [14,15] and inductively coupled plasma mass spectrometry (ICP-MS) [16] offer high accuracy but require expensive equipment and complex sample preparation, making them unsuitable for in situ and in vivo analysis. In this context, fluorescence spectroscopy stands out due to its high sensitivity, simplicity, speed of analysis, real-time visualization capabilities, and low cost [17].

Among the various fluorescent platforms (chelates, nanoparticles, polymers, etc.), Schiff bases, particularly hydrazones, have proven to be extremely flexible and efficient sensor nodes [18,19]. A hydrazone (-CH=N-NH-C=O) is an azomethine fragment formed by the condensation of a hydrazide with an aldehyde or ketone. Its popularity in chemosensorics is attributed to the simplicity of synthesis, the ability to chelate metals through the nitrogen and oxygen atoms of the carbonyl group, and the capacity to fine-tune photoluminescent properties by introducing various fluorescent fragments [20].

Schiff bases and hydrazones derived from vitamin B_6_ exhibit excellent chelating abilities, are soluble in water, and possess low toxicity, which makes them appealing for the design of fluorescent chemosensors and probes suitable for in vivo applications [21]. Over the last ten years, a variety of fluorescent probes featuring a vitamin B_6_ component have been designed to detect cations in both aqueous and water-organic mixtures, including Cu^2+^ [22], Zn^2+^ [23,24,25], Cd^2+^ [26], Ba^2+^ [27], Al^3+^ [28] and Ga^3+^ [26,29].

Currently, there is active progress in the development of fluorescent probes for detecting gallium(III) and aluminum(III) ions using a range of organic compounds, supported by several successful studies. Fluorescent probes based on naphthalene [30,31,32], quinoline [33], coumarin [34], vitamin B_6_ [26,29], and rhodamine [35] derivatives have been developed for the determination of Al^3+^ and Ga^3+^ ions. Therefore, the design of fluorescent probes that can selectively identify Al^3+^ and Ga^3+^ ions represents a significant and timely field of research.

In this work we prepared a simple fluorescence probe (probe **1**) based on vitamin B_6_ and furan-2-carbohydrazide for selective and sensitive recognition of Al^3+^ and Ga^3+^ ions in buffered DMSO. The influence of a wide range of metal cations (Na^+^, K^+^, Ca^2+^, Mg^2+^, Ba^2+^, Co^2+^, Ni^2+^, Cu^2+^, Zn^2+^, Cd^2+^, Pb^2+^, Hg^2+^, Cr^3+^, Fe^3+^, Ce^3+^, Al^3+^, In^3+^) on the determination of Al^3+^ and Ga^3+^ ions in solution has been studied in detail. In addition, quantum chemical calculations of the geometric and energy parameters for probe **1**, as well as for complexes **1**-Al^3+^ and **1**-Ga^3+^, were carried out.

## 2. Materials and Methods

### 2.1. Chemicals

Pyridoxal 5′-phosphate (from abcr, Karlsruhe, Germany), furan-2-carbohydrazide (from Sigma-Aldrich, St. Louis, MO, USA), and tris(hydroxymethyl)aminomethane (also from Sigma-Aldrich, USA) were utilized without further purification, with a reagent purity exceeding 98%. Metal cations, including Na^+^, K^+^, Ca^2+^, Mg^2+^, Ba^2+^, Co^2+^, Ni^2+^, Cu^2+^, Zn^2+^, Cd^2+^, Pb^2+^, Hg^2+^, Cr^3+^, Fe^3+^, Ce^3+^, Al^3+^, Ga^3+^, and In^3+^ (sourced from Reakhim, Staraya Kupavna, Russia), were obtained as nitrate salts and used as received. Anions such as F^−^, Cl^−^, Br^−^, I^−^, NCS^−^, NO_3_^−^, H_2_PO_4_^−^, HSO_4_^−^, CN^−^, ClO_3_^−^, and ClO_4_^−^ were also acquired from Reakhim, Russia, in the form of sodium or potassium salts and employed without purification. Solutions of the cation and anion salts were prepared using double-distilled water (*κ* = 3.6 μS/cm, pH = 6.6). Dimethyl sulfoxide (>99.9%; EKOS-1, Staraya Kupavna, Russia) was used as purchased as well.

### 2.2. Synthesis of the Probe 1

**Figure 1:**
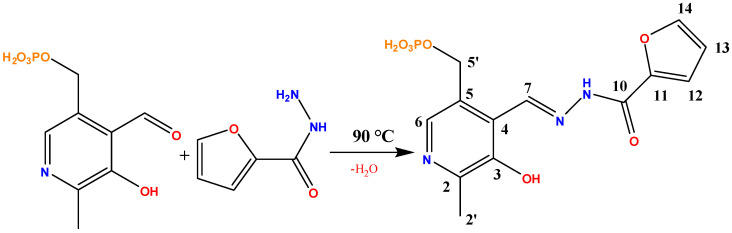
Synthesis of the probe **1** with atom numbering.

The synthesis of probe **1** was adapted from literature [36], with the reaction scheme depicted in Figure 1. A solution of pyridoxal-5′-phosphate (247 mg, 1 mmol) in water (20 mL) was heated to 90 °C and treated with an aqueous solution (15 mL) of furan-2-carbohydrazide (126 mg, 1 mmol). Upon cooling to room temperature over 1 h, a pale yellow precipitate formed. The solid was isolated by filtration, washed sequentially with cold distilled water and acetone, and dried to yield the desired hydrazone (298 mg, 84%). Comprehensive characterization by ^1^H, ^13^C and ^31^P NMR, IR spectroscopy, and mass spectrometry confirmed the successful synthesis and high purity of the product (see supporting data).

**(E)-(4-((2-(furan-2-carbonyl)hydrazono)methyl)-5-hydroxy-6-methylpyridin-3-yl)methyl dihydrogen phosphate** (probe **1**): ^1^H NMR, 500.17 MHz, *δ*, ppm (DMSO-*d6*): 12.76 s (1H, OH), 12.30 s (1H, NH), 8.87s (1H, H_7_), 8.02 s (2H, H_6_, H_14_), 7.41 d (^3^*J* = 3.5 Hz, 1H, H_12_), 6.76 d (^3^*J* = 3.5 Hz, H_13_), 5.05 d (^3^*J* = 8.2 Hz, 2H, H_5′_), 2.44 s (3H, H_2′_). ^13^C NMR, 125.77 MHz, *δ*, ppm (DMSO-*d6*): 154.6 (C_3_), 151.1 (C_2_), 148.9 (C_7_), 147.1 (C_14_), 146.2 (C_11_), 146.0 (C_6_), 139.6 (C_5_), 128.5 (C_4_), 116.7 (C_12_), 112.9 (C_13_), 62.8 (C_5′_), 19.3 (C_2′_). ^31^P NMR, 202.47 MHz, *δ*, ppm (DMSO-*d6*): −1.53 s. IR(KBr), cm^−1^: 3418 w, 3256 w, 3113 w, 2923 m, 2854 w, 1696 s, 1576 s, 1540 s, 1478 s, 1354 m, 1270 s, 1231 m, 1178 s, 1087 s, 1019 m, 987 m, 925 m, 847 m, 753 w, 639 w, 600 w. *m*/*z* = 356.79 [**1**-H^+^], theoretical *m*/*z* = 356.25 [**1**-H^+^]. IR, MS, ^1^H, ^13^C and ^31^P NMR spectra are given in the Supplementary information (Figures S1–S5).

### 2.3. Spectral Measurements

The UV-Vis absorption spectra of probe **1** (50 µM), both free and in the presence of various equimolar metal ions (Na^+^, K^+^, Ca^2+^, Mg^2+^, Ba^2+^, Co^2+^, Ni^2+^, Cu^2+^, Zn^2+^, Cd^2+^, Pb^2+^, Hg^2+^, Cr^3+^, Fe^3+^, Al^3+^, Ga^3+^, In^3+^ and Ce^3+^), were acquired using a Shimadzu UV1800 double-beam spectrophotometer (Shimadzu, Canby, OR, USA). Measurements were conducted at 298.2 ± 0.1 K in a DMSO/0.01 M Tris-HCl buffer mixture (9:1 *v*/*v*, pH 7.4), using the solvent mixture as a blank. The spectra were recorded from 240 to 500 nm with an absorbance range of 0–2. The instrument’s wavelength and absorbance accuracies were within 0.5 nm and ±0.006 units, respectively.

All fluorescence spectra were recorded on a Shimadzu RF6000 spectrofluorometer (Shimadzu, Canby, OR, USA) with the excitation and emission slit widths set to 5 nm. The fluorescence spectra of probe **1** (50 µM) and its mixtures with equimolar cations (50 µM) were measured in the same solvent system using an excitation wavelength of 411 nm, with emission recorded from 430 to 650 nm. All fluorescence experiments were performed at 298.2 ± 0.1 K. The quantum yield of **1**, **1**+Al^3+^, **1**+Ga^3+^ was determined using the relative method [37] with quinine sulfate in 0.5 M H_2_SO_4_ (Φ_f,st_ = 0.546) as the standard [38].

Mass spectrometry (MS) analysis was performed on a Shimadzu Biotech Axima Confidence MALDI-TOF mass spectrometer (Shimadzu, Carlsbad, CA, USA). Samples were prepared by applying an ethanolic solution of probe **1** (0.05 mmol) to the target plate and allowing it to dry under ambient conditions.

Infrared (IR) spectra were obtained using a Thermo Nicolet Avatar 360 FTIR spectrometer (Thermo Nicolet, Sugarland, TX, USA). Measurements were performed in the range of 400–4000 cm^−1^ using potassium bromide (KBr) pellets.

NMR experiments were conducted on probe **1**, as well as on mixtures of **1**-Al^3+^ and **1**-Ga^3+^ in DMSO-*d_6_*, using a Bruker Avance III 500 NMR spectrometer (Bruker, Mannheim, Germany), with ^1^H, ^13^C and ^31^P operating frequencies of 500.17 MHz, 125.77 MHz, and 202.47 MHz, respectively. Temperature regulation was maintained with a Bruker variable temperature unit (BVT-2000).

### 2.4. Quantum Chemical Calculations

The molecular structures of probe **1** and **1**-Al^3+^ and **1**-Ga^3+^ were modeled for the singlet electronic state with Gaussian 09W [39]. Using the CAM-B3LYP hybrid DFT method [40], their geometric properties, vibrational frequencies, vertical transition energies, GIAO-calculated shielding constants and UV-Vis oscillator strengths were computed. All atoms (H, C, N, O, P, Al, and Ga) were treated with the cc-pVTZ full-electron basis set [41], and DMSO solvation effects were included via the polarizable continuum model (PCM) [42]. Solvent and buffer molecules (DMSO, H_2_O, and Tris) that may be present in the complex were not considered in the calculation. The calculations of system **1**-Al^3+^ and **1**+Ga^3+^ had been done in the framework +3e charge value. Visualizations of molecules and orbitals were created with Chemcraft 1.8 [43]. Atomic charges according to Bader’s theory [44] and molecular electrostatic potentials (MEP) were obtained using the AIMAll software (Version 19.10.12) [45].

## 3. Results and Discussion

### 3.1. Experimental Study

**Figure 2:**
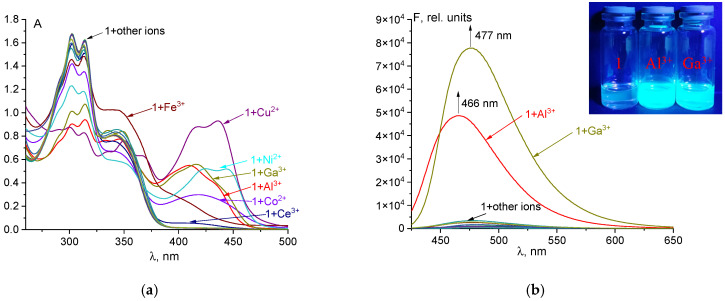
UV-Vis spectra (**a**) and fluorescence spectra (**b**) of the probe **1** (50 µM) and its mixtures with different cations (1 equivalent) Insert: naked eye fluorescence of **1**, **1**+Al^3+^, **1**+Ga^3+^ in DMSO/0.01 M Tris-HCl buffer mixture (9:1 *v*/*v*, pH 7.4), λ_ex_ = 365 nm (UV lamp).

The response of probe **1** to various ions was initially investigated using UV-Vis and fluorescence spectroscopy in a DMSO/Tris-HCl buffer solution (9:1, *v*/*v*, pH 7.4). Tris-HCl buffer was used to maintain a constant pH in a mixed aqueous-organic solution, as vitamin B_6_ hydrazones are pH-sensitive [46], and pH fluctuations could introduce errors in metal ion detection. As shown in Figure 2a, the free probe **1** displays a double peak at 302 and 314 nm, along with a characteristic absorption shoulder at 340 nm, which is consistent with reported spectra for vitamin B_6_ hydrazones in neutral media [24,28]. Upon addition of Ni^2+^, Co^2+^, Cu^2+^, Ce^3+^, Fe^3+^, Al^3+^, or Ga^3+^ ions, the solution changed from colorless to yellow, accompanied by bathochromic shifts in the UV-Vis spectra. The distinct spectral shapes for the different metal complexes suggest that the electronic structure of the metal ion influences the complex’s photophysical properties. Nevertheless, these results indicate that UV-Vis spectroscopy alone is insufficient for the definitive determination of specific metal cations. Probe **1** exhibits weak fluorescence at ~478 nm in a buffered DMSO solution. This low quantum yield is attributed to the molecule’s labile structure, where distortion in the excited state promotes non-radiative decay to the ground state.

In addition, fluorescence can be suppressed due to photoinduced electron transfer (PET) [47] from donor nitrogen atoms to vitamin B_6_, which provides a non-radiative pathway for relaxation of the excited state. Complexation with open-shell d-metal ions (e.g., Ni^2+^, Co^2+^, Cu^2+^, and Fe^3+^) quenches the fluorescence. This occurs due to the metals’ low-energy triplet d-d states, which facilitate non-radiative deactivation pathways, primarily through intersystem crossing and internal conversion. In contrast, the addition of Al^3+^ and Ga^3+^ ions results in a significant fluorescence enhancement, visible to the naked eye (insert Figure 2b). The emission maxima for the resulting complexes are located at 466 nm for Al^3+^ and 477 nm for Ga^3+^ (Figure 2b). Consequently, the fluorescence quantum yield increases substantially from 0.4% to 8.4% for Al^3+^ and 13.8% for Ga^3+^. These results demonstrate that probe **1** can be utilized for the fluorimetric detection of aluminum(III) and gallium(III) ions in aqueous DMSO media.

**Figure 3:**
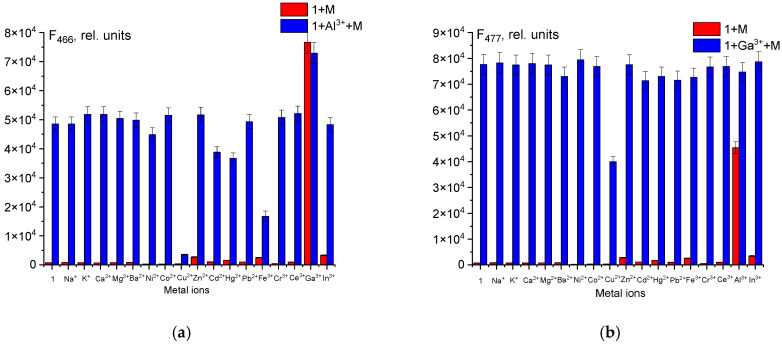
The fluorescence intensity of probe **1** (50 µM) upon addition of various metal ions (50 µM) in the absence/presence of Al^3+^ (**a**) and Ga^3+^ (**b**). (Red bars: **1** with various metal ions. Blue bars: **1** with various metal ions in the presence of Al^3+^ (**a**) and Ga^3+^ (**b**).

**Figure 4:**
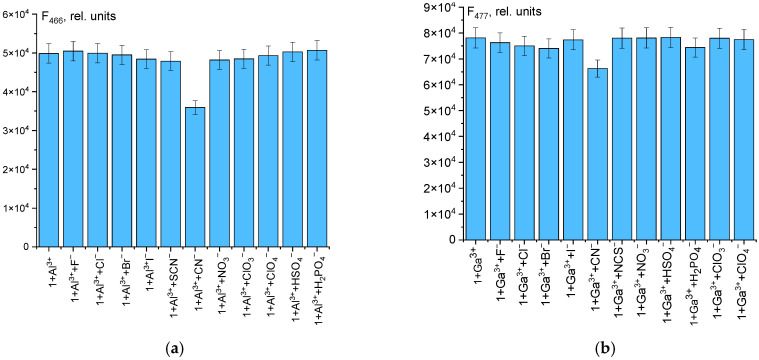
The fluorescence intensity of probe **1** (50 µM) upon addition of various anions (50 µM) in the presence of Al^3+^ (**a**) and Ga^3+^ (**b**) in a DMSO/0.01 M Tris-HCl buffer mixture (9:1 *v*/*v*, pH 7.4).

Effective fluorescent probes for detecting metal ions must exhibit high selectivity and sensitivity toward the target ion in the presence of competing species. To evaluate the selectivity of probe **1** for Al^3+^ and Ga^3+^, we investigated its fluorescence response in the presence of various competing cations and anions in a buffered dimethyl sulfoxide solution (Figure 3 and Figure 4). The probe **1** demonstrated a higher affinity for Ga^3+^ over Al^3+^. Among the competing ions, only Cu^2+^ caused significant interference, quenching the fluorescence intensity by approximately 50%. For aluminum(III) determination, ions of alkaline and alkaline earth metals, as well as Zn^2+^, Pb^2+^, Cr^3+^, and In^3+^, showed negligible effects. In contrast, Cu^2+^ and Fe^3+^ are major interfering cations, as they form stable non-fluorescent complexes with probe **1**, effectively displacing Al^3+^ and precluding its accurate detection. Consequently, probe **1** is suitable for the qualitative determination of Ga^3+^, but not Al^3+^, in the presence of Cu^2+^. Copper(II) ions can be masked with a solution of ammonia or cyanide ions by converting them into stable complex compounds that do not react with probe **1** [48]. Furthermore, in mixtures containing both Al^3+^ and Ga^3+^, the probe responds selectively, allowing only for the determination of Ga^3+^. The high affinity for Ga^3+^ is likely attributable to its stronger Lewis acidity compared to Al^3+^, which complements the size of the hydrazone’s coordination cavity.

The probe **1** demonstrates excellent selectivity for Al^3+^ and Ga^3+^ over a wide range of competing anions. No significant interference was observed from halides, oxyanions, or other common anions. An interference was noted only for CN^−^, which quenches the fluorescence by ≈30% and ≈15% for Ga^3+^ (Figure 4). This quenching is presumably a result of cyanide coordinating with cations to form stable complexes like [M(CN)_4_]^−^ or [M(CN)_6_]^3−^ [49], thereby competing with the probe for the metal ion. In summary, while probe **1** exhibits high selectivity for its target cations (Al^3+^ and Ga^3+^) against most background ions, the use of masking agents is recommended for analyses in complex systems containing interfering ions like Cu^2+^ and Fe^3+^.

**Figure 5:**
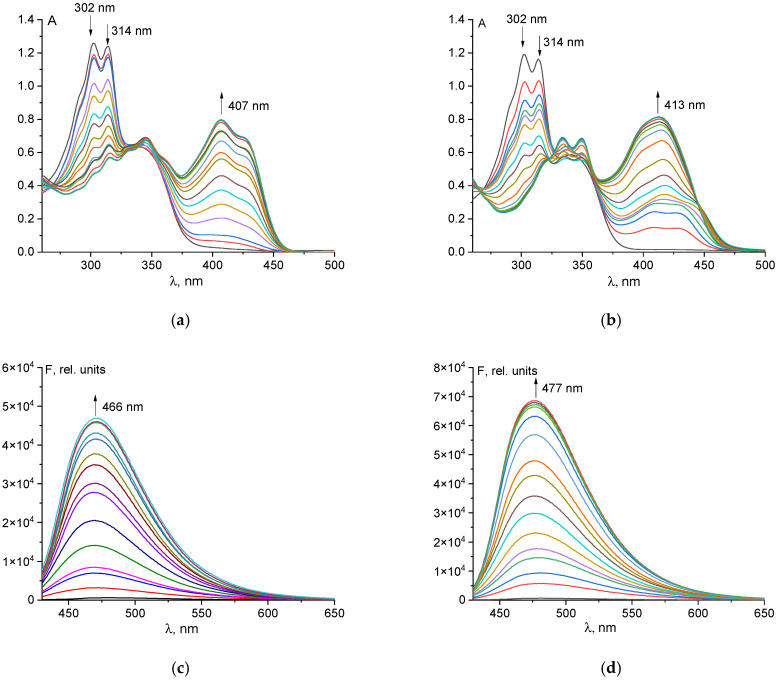
UV-Vis spectral changes in the probe **1** (50 µM) upon the addition of Al^3+^ (**a**) and Ga^3+^ ions (**b**); fluorescence spectral changes in the probe **1** (50 µM) upon the addition of Al^3+^ (**c**) and Ga^3+^ ions (**d**).

The UV–Vis spectra of probe **1** upon addition of aluminum and gallium ions are presented in Figure 5a,b. Following the introduction of Al^3+^ and Ga^3+^, the absorbance maxima at 302 nm and 314 nm gradually diminish. Concurrently, a high-intensity peak emerges at 407 nm for aluminum(III) and 413 nm for gallium(III). The shape of the long-wavelength absorption maximum differs for the aluminum and gallium complexes, indicating a distinct influence of the central ion on the frontier orbitals of probe **1**. This long-wavelength maximum can be attributed to charge transfer within the coordination complex. Charge transfer transitions in UV–Vis spectroscopy are typically associated with ions possessing high oxidation states [50]. The stability constants and stoichiometry of the complexes formed between probe **1** and Al^3+^/Ga^3+^ ions were determined using the KEV software, ver. 0.6.0 [51]. The 1:1 binding model yielded standard deviations for the stability constant that were similar to those of the 1:2 model (0.28 vs. 0.29 for Al^3+^, 0.43 vs. 0.37 for Ga^3+^), and comparable R^2^ values (0.989 vs. 0.977 for Al^3+^, 0.977 vs. 0.961 for Ga^3+^) (Figures S6 and S7). However, the calculated UV-Vis spectra for the 1:1 complexes agree well with the experimental data, whereas the 1:2 model produced nonphysical negative extinction coefficients in the 280–330 nm range (Figures S8 and S9). Therefore, for both metal ions, the 1:1 binding model describes the data more reliably than the 1:2 binding model. It is worth noting that the stability constants are quite high (log *β′* = 5.98 for **1**-Al^3+^ and log *β′* = 6.28 for **1**-Ga^3+^), indicating a strong affinity for aluminum(III) and gallium(III) ions. The higher stability constant with gallium is likely attributable to the structure of the vitamin B_6_-derived hydrazone chelating cavity. The larger ionic radius of gallium may enable more efficient interaction with the donor atoms of probe **1**. The sensitivity of probe **1** to Al^3+^ and Ga^3+^ ions was evaluated by fluorescence titration in a DMSO/Tris-HCl buffer solution (9:1, *v*/*v*, pH 7.4) (Figure 5c,d). Upon gradual addition of Al^3+^ and Ga^3+^, the fluorescence intensity of probe **1** increased significantly. The maximum emission was observed at 466 nm for Al^3+^ and at 477 nm for Ga^3+^. Notably, the gallium(III) complex also exhibited absorption at longer wavelengths. The observed Stokes shift of ~60 nm is moderate and typical for many fluorophores [17]. The corresponding Stokes shifts were 59 nm for the aluminum(III) complex and 64 nm for **1**-Ga^3+^. Plots of fluorescence intensity at the emission maximum versus metal ion concentration were linear up to 45 µM for Al^3+^ and 35 µM for Ga^3+^, enabling the quantitative determination of these ions in samples.

**Figure 6:**
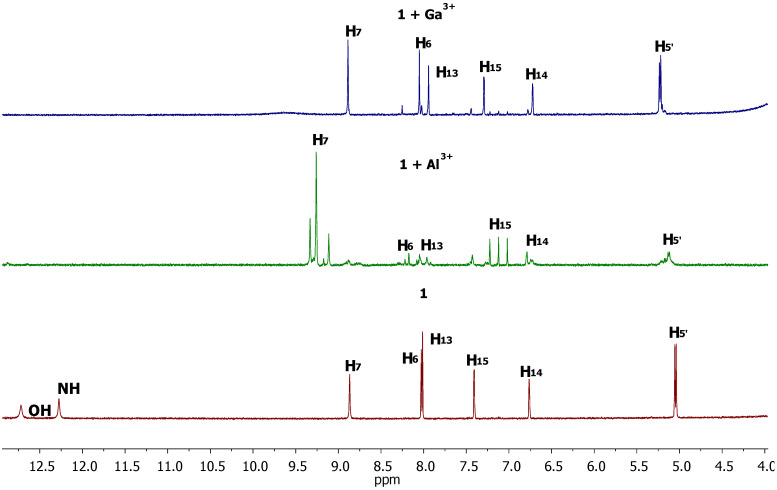
^1^H NMR experiment of probe **1** with Al^3+^ and Ga^3+^ in DMSO-*d_6_*. The atoms are numbered according to Figure 1.

To gain deeper insight into the characteristics of the probe coordination compound formed with Al^3+^ and Ga^3+^ ions, a ^1^H NMR experiment was conducted. Probe **1** was dissolved in DMSO-*d_6_*, and metal ions were introduced into separate NMR tubes (Figure 6). The ^1^H NMR spectroscopic analysis provides critical insights into the coordination behavior of probe **1** with Al^3+^ and Ga^3+^ ions. The observed signal splitting upon metal ion addition is characteristic of a slow exchange regime on the NMR timescale [52,53]. This suggests that the complexes formed are kinetically inert, with the strong electrostatic interaction stemming from the high charge density of Al^3+^ and Ga^3+^ hindering the dissociation of the ligand. Such behavior is consistent with the formation of stable metal-centered complexes. A notable difference between the two metal ions is the greater degree of signal splitting observed for the Al^3+^ complex compared to the Ga^3+^ complex. This distinction likely reflects differences in their ionic radii, charge density, and coordination preferences. Al^3+^, being smaller, may form a more rigid complex or engage in additional coordination interactions that are not accessible to Ga^3+^. Specifically, the data suggest that Al^3+^ may participate in an additional binding mode involving the phosphate group of probe **1**. This hypothesis is supported by the distinct chemical shift changes observed for the methylene (H_5′_) protons adjacent to the phosphate moiety. The fact that the phosphate group is electronically isolated from the conjugated system by a methylene spacer explains why this secondary binding event does not translate into observable changes in the absorption or fluorescence spectra, allowing for a dissociation between the structural and optical responses. The downfield shift in proton signals upon complexation for both metals supports an electronic effect wherein coordination to the metal center reduces the electron density around the hydrazone framework, consistent with a decrease in shielding. This is typical for complexes formed with Lewis acidic metal ions. Overall, the NMR evidence points to a primary coordination site for both Al^3+^ and Ga^3+^ within the hydrazone moiety, with an additional, secondary interaction involving the phosphate group in the case of Al^3+^.

**Figure 7:**
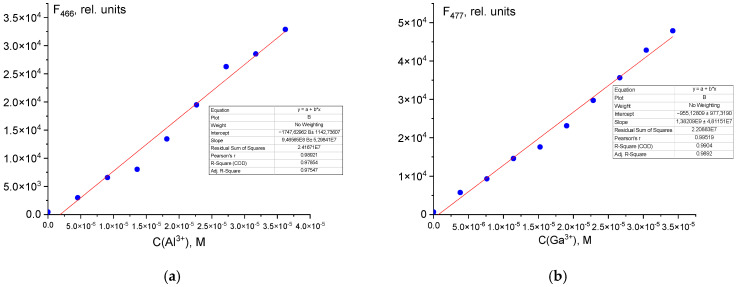
Limit of detection of Al^3+^ (**a**) and Ga^3+^ (**b**) ions.

The limits of detection (LOD) and quantification (LOQ) for Al^3+^ and Ga^3+^ using probe **1** were calculated based on the 3*σ* rule [54], yielding values of 0.048 µM (LOD) and 0.16 µM (LOQ) for Al^3+^, and 0.033 µM (LOD) and 0.11 µM (LOQ) for Ga^3+^ (Figure 7). The hydrazone derived from pyridoxal-5-phosphate and hydrazide 2-furancarboxylic acid therefore serves as a highly sensitive probe for these ions. The obtained LOD values are competitive with those reported for other probes in the literature (Tables S1 and S2). Notably, probe **1** is among the most sensitive probes described while also featuring a simple, reproducible synthesis with high yield, an important practical advantage for potential applications such as ion detection in environmental and biological samples.

**Figure 8:**
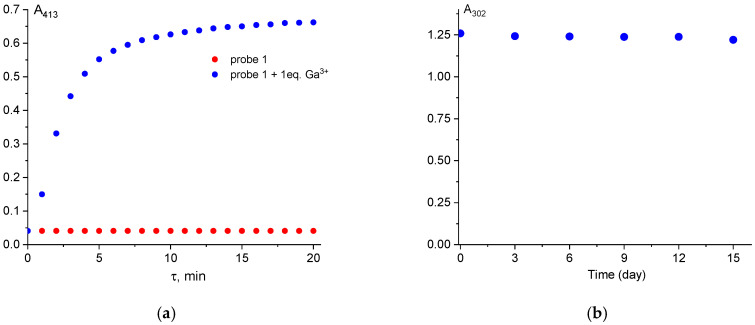
The influence of response time on the absorbance intensity of the probe **1** + Ga^3+^ (1 eq.) in DMSO/Tris-HCl, pH 7.4 (9:1 *v*/*v*) (**a**); Absorption change in the probe **1** in 2 weeks (**b**).

The probes must exhibit fast ion detection kinetics and stability in solution, as these properties are critical for practical applications. The response time of probe **1** toward Al^3+^ and Ga^3+^ was investigated by UV-Vis spectroscopy (Figure 8a). In the absence of metal ions, the absorbance of probe **1** at 413 nm remained constant over 20 min (red points). Upon addition of Al^3+^, the absorbance reached its maximum within 10 min and then remained stable (blue points). These results demonstrate that probe **1** can detect gallium(III) ions rapidly and efficiently. In contrast, the reaction between probe **1** and Al^3+^ was considerably slower (Figure S10). To ensure the reaction reaches equilibrium, it is recommended that the solutions be left overnight prior to measurement. Furthermore, the absorbance maximum of probe **1** remained unchanged for two weeks, indicating excellent stability of the hydrazone derivative in DMSO/Tris-HCl, pH 7.4 (9:1 *v*/*v*) solution (Figure 8b).

**Figure 9:**
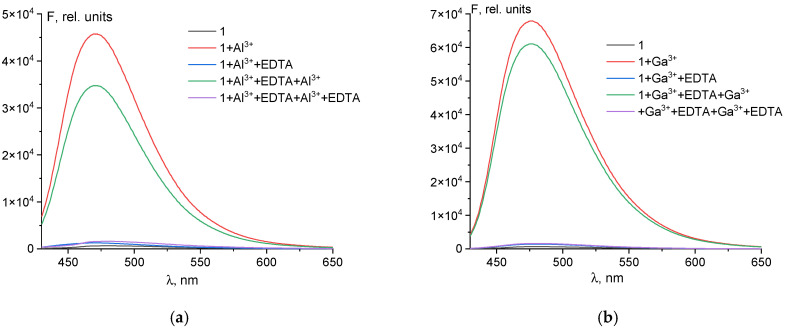
Reversibility of probe **1** (50 μM) with subsequent addition of 1 equivalent of Al^3+^ (**a**) or Ga^3+^ (**b**) and Na_2_EDTA (50 μM) in DMSO/Tris-HCl, pH 7.4 (9:1 *v*/*v*).

An important factor for the application of probe **1** under realistic conditions is its reversibility. This was effectively demonstrated in experiments with EDTA, as shown in Figure 9. The obtained results confirm that probe **1** is capable of performing repeated analyses for Al^3+^ and Ga^3+^ ions in samples. The decrease in fluorescence intensity upon repeated addition of Al^3+^ or Ga^3+^ can be attributed to experimental error in the ratio of metal ion to EDTA concentrations, as well as possibly to dilution of the solution.

### 3.2. DFT Calculations

**Figure 10:**
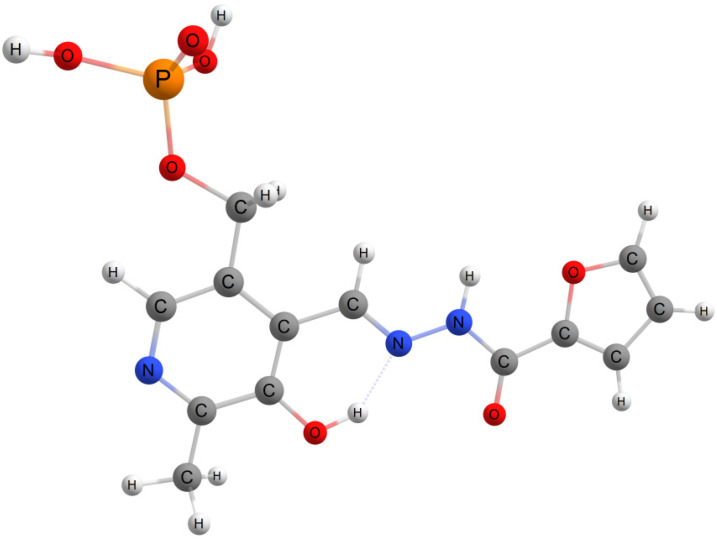
Molecular structure of probe **1**.

Understanding the molecular structure of chemical probes enables the design of analytical techniques with enhanced sensitivity and selectivity. Figure 10 depicts the structure of probe **1**, which features a planar, conjugated framework. The conformation is stabilized by an intramolecular hydrogen bond between the phenolic hydroxyl group and the azomethine nitrogen. Furthermore, the furan fragment relative to the hydrazone backbone is also stabilized by a hydrogen bond formed between the N-H proton and the heterocyclic oxygen atom.

**Figure 11:**
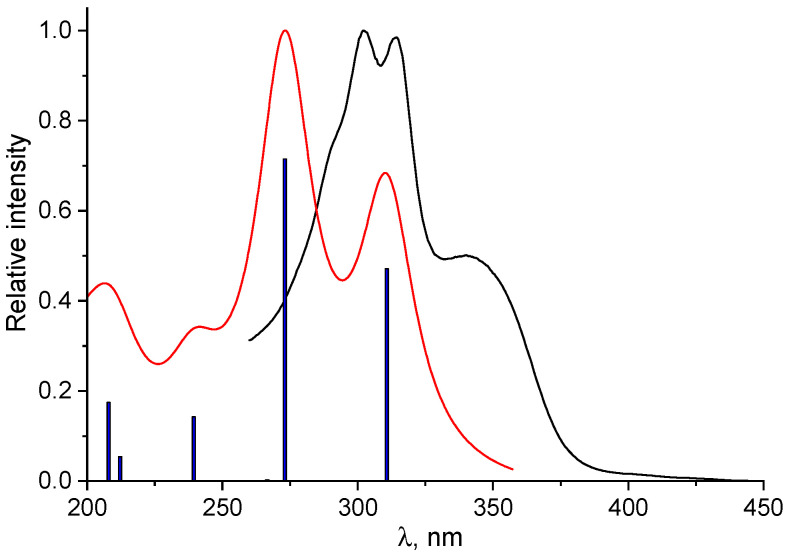
Normalized calculated electronic absorption spectrum for probe **1** (red line) with comparison with experimental (black line). Blue lines represent the oscillator strengths of the electronic transitions.

Figure 11 compares the experimental and TDDFT-calculated electronic spectra of probe **1**. The theoretical spectrum demonstrates good agreement with the experimental data, showing a minor hypsochromic shift of about 31 nm in the absorption maximum (273 nm calculated vs. 304 nm experimental). The longest wavelength transition responsible for the arm at 340 nm in the experimental spectrum is associated with the S_0_→S_1_ excitation. This transition is mainly (95%) described by excitation from HOMO to LUMO. While the HOMO is largely confined to the conjugated π-system of the vitamin B_6_ moiety and the hydrazone bridge with minimal furan involvement.

The contribution of the furan fragment increases significantly in the LUMO. In this orbital, the electron density is delocalized over the π* system of the hydrazone. It is important to note that the phosphate group remains uninvolved in these frontier molecular orbital transitions due to its electronic decoupling from the main chromophore, achieved via an intervening methylene (-CH_2_-) spacer. Analysis of the primary absorption band reveals that it originates from the S_0_→S_2_ transition. This electronic excitation is characterized by a major configuration (82%) corresponding to HOMO-1→LUMO and a secondary one (10%) from HOMO-2→LUMO. The HOMO-2 orbital, one of the contributors, exhibits electron density delocalized over most of the probe’s framework, with the notable exclusion of the phosphate and methyl substituents. Furthermore, the HOMO-1 orbital differs from HOMO-2 by also showing negligible density on the carbonyl moiety. It is notable that the molecular orbitals of the phosphate group contribute to the high-energy S_0_→S_7_ and S_0_→S_7_ transitions, which are located in the 200–240 nm region. The compositions of the lowest excited states, corresponding oscillator strengths, and visual representations of the molecular orbitals for probe **1** are provided in Tables S3 and S4.

**Figure 12:**
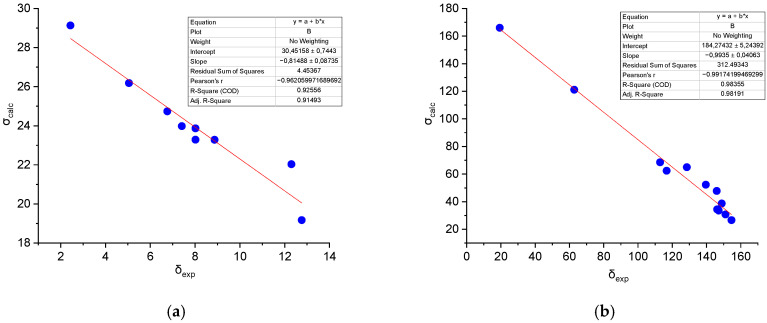
Correlation between experimental ^1^H (**a**) and ^13^C (**b**) chemical shifts and GIAO shielding constants for probe **1**.

The experimental ^1^H and ^13^C chemical shifts in probe **1** were correlated with their corresponding GIAO-calculated shielding constants (Figure 12). The calculated GIAO screening constants show satisfactory agreement with the experimental chemical shifts. Good correlation is observed for the majority of proton and carbon signals in probe **1**, allowing for clear assignment of signal types.

For instance, the methyl group protons, which are the most shielded, correspond to the region of highest electron density. In contrast, deshielded protons such as O-H and N-H, as well as carbons bonded to oxygen or nitrogen atoms, appear in the predicted lower-field regions due to the electron-withdrawing effect of these electronegative atoms. Some deviations between calculated and experimental values were noted. The most significant discrepancy occurs for the N-H proton signal in the ^1^H NMR spectrum. This is likely attributable to the absence of explicit solvent modeling in the calculations and limitations of the chosen basis set. Additionally, the signal for the carbonyl carbon (C=O) could not be observed in the experimental ^13^C NMR spectrum due to the low solubility of probe **1** in DMSO-*d6*.

The molecular structures of the **1**-Al^3+^ and **1**-Ga^3+^ complexes are shown in Figure S11. Probe **1** acts as a tridentate ligand, coordinating to the metal ions via the phenolic oxygen, azomethine nitrogen, and carbonyl oxygen atoms. Key bond distances for the free probe and its Al^3+^ and Ga^3+^ complexes are listed in Table 1. The complexation of probe **1** with Al^3+^ and Ga^3+^ induces a distinct geometric rearrangement of the hydrazone moiety. This is manifested by an elongation of most bonds, contrasting with a contraction of the carbonyl carbon–nitrogen (C-N) bond. Comparing the two metal complexes, the M-O and M-N bond lengths are longer in the **1**-Ga^3+^ complex than in the **1**-Al^3+^ analog (Table 1), a direct consequence of the larger ionic radius of Ga^3+^. Notably, in both complexes, the M–O bond is shorter than the M–N bond. This structural feature highlights the greater binding affinity of these trivalent cations for the hard oxygen donor site, aligning with the Pearson hard acid character of Al^3+^ and Ga^3+^ and the hard base nature of the phenolate oxygen.

**Figure 13:**
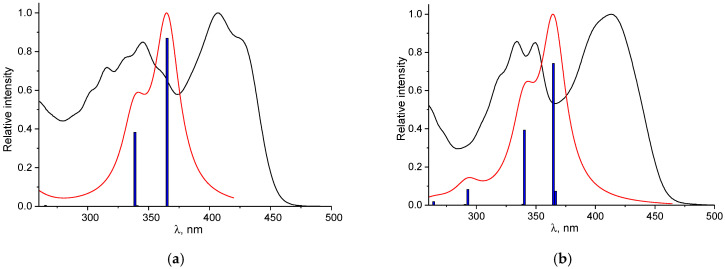
Normalized calculated electronic absorption spectrum for **1**–Al^3+^ and **1**–Ga^3+^ (red line) with comparison with experimental (black line). Blue lines represent the oscillator strengths of the electronic transitions.

A comparison between the experimental UV-Vis spectra of **1**–Al^3+^ and **1**–Ga^3+^ and calculated TDDFT is presented in Figure 13. The theoretical spectra demonstrate good agreement with the experimental data; a hypsochromic shift in the absorption maximum by 42–48 nm is demonstrated during the transition from experimental to theoretical spectra. While the experimental and calculated electronic spectra of the Al^3+^ and Ga^3+^ complexes show good agreement, their underlying electronic transitions and orbital compositions differ significantly. In the **1**–Al^3+^ complex, the intense lowest-energy S_0_→S_1_ transition arises primarily (73%) from a HOMO→LUMO excitation. The HOMO is dominated by contributions from the furan and hydrazone π-system, with a minor role from the vitamin B_6_ moiety, whereas the LUMO is largely localized on the vitamin B_6_ unit and the hydrazone bridge, with negligible involvement from the Al^3+^ ion and furan. In stark contrast, the corresponding S_0_→S_1_ transition in the **1**–Ga^3+^ complex at 404 nm is of very low intensity (oscillator strength = 0.0005), despite having an even larger HOMO→LUMO contribution (93%).

Here, the HOMO retains a similar ligand-centered character (furan/hydrazone) as **1**-Al^3+^, but the LUMO is predominantly localized on the gallium(III) ion and the coordinating nitrogen and oxygen atoms. This orbital distribution is characteristic of a ligand-to-metal charge transfer (LMCT) transition. This fundamental difference in electronic structure can be rationalized by the electronic configurations of the metal ions. Unlike Al^3+^, which has a closed-shell [Ne] configuration with no available low-energy d-orbitals, Ga^3+^ possesses energetically accessible 3d-orbitals. These vacant d-orbital domains provide an acceptor site for LMCT transitions, thereby significantly altering the nature of the lowest unoccupied molecular orbital and the photophysics of the complex. The most intense transition for **1**-Ga^3+^, S_0_→S_3_, calculated at a wavelength of 365 nm, corresponds to the intense experimental absorption band observed at 413 nm. It is dominated by the HOMO→LUMO+1 excitation (66%), with a significant contribution from HOMO-1→LUMO+1 (23%). The HOMO-1 orbital is delocalized on the pyridine moiety of vitamin B_6_, whereas the LUMO+1 is delocalized across the entire chromophore, with a small contribution from the Ga^3+^ ion. The compositions of the lowest excited states, corresponding oscillator strengths, and visual representations of the molecular orbitals for probe **1**-Al^3+^ and **1**-Ga^3+^ are provided in Tables S3 and S4.

**Figure 14:**
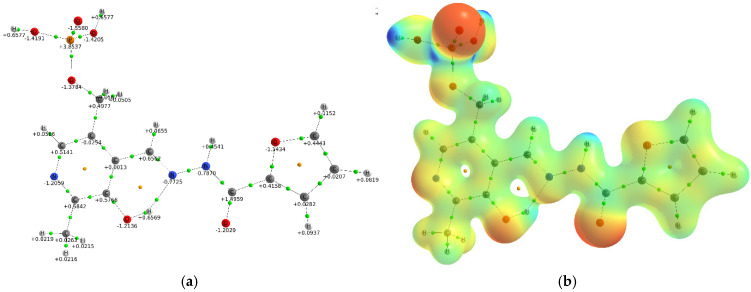
Molecular graph with atomic charges for probe **1** (**a**). The bond critical points are green, ring critical points are yellow. The molecular electrostatic potential (MEP) mapped on the isodensity surface (0.04 a.u.) for probe **1** (**b**).

The Atoms in Molecules (AIM) methodology provides a robust framework for investigating non-covalent interactions, such as hydrogen bonds, through the analysis of electron density properties at bond critical points (BCPs). These closed-shell interactions are identified by distinct features in the electron density and its Laplacian. Figure 14 presents the molecular graph and molecular electrostatic potential (MEP) for probe **1**. The molecular graph indicates an intramolecular hydrogen bond between the hydroxyl proton of vitamin B_6_ and the azomethine nitrogen. This interaction is validated by AIM analysis, which yields a BCP electron density of −0.0478 a.u., corresponding to an interaction energy of −15.00 kcal/mol [55]. The strength of this O–H⋯N bond in probe **1** is approximately double that observed in a related hydrazone derived from 5-chlorosalicylaldehyde and 2,4-dinitrophenylhydrazide [56]. This increased stability is likely due to the electron-withdrawing nitro groups, which enhance the electron deficiency of the azomethine nitrogen via a negative mesomeric effect. On the other hand, substituting furan for pyrazine in vitamin B_6_ hydrazone has practically no effect on the strength of the O–H⋯N hydrogen bond (−15.00 vs. −14.83 kcal/mol) [28]. The MEP reveals the most electronegative regions (depicted in red) localized on the nitrogen and oxygen atoms, consistent with the presence of lone electron pairs. Conversely, the most electropositive potentials (depicted in blue) are found on the hydrogen atoms of the phosphate group and the N-H proton of the hydrazone moiety. Structural analysis of probe **1** indicates four potential sites for metal ion coordination: (i) the pyridine nitrogen, (ii) the phosphate group, (iii) a chelating cavity formed by the phenolic oxygen, azomethine nitrogen, and carbonyl oxygen atoms, and (iv) the furan oxygen. Notably, the furan oxygen is a weak o-donor, as its lone pairs are partially delocalized within the aromatic ring [57]. Furthermore, a methylene spacer separates the phosphate group from the primary hydrazone chromophore. Upon complexation with aluminum(III) and gallium(III) ions, a bathochromic shift in the UV-Vis spectrum is observed (Figure 5).

This spectroscopic change suggests that the phosphate group likely does not participate in coordinating these particular ions. Instead, the tridentate chelating cavity (phenolic O, azomethine N, carbonyl O) appears to be the preferred coordination site for Al^3+^ and Ga^3+^. The presence of three donor atoms within this cavity is expected to confer greater stability to the resulting complexes compared to those formed with mono- or bidentate ligands. In probe **1**, a greater effective negative charge is concentrated on the oxygen and nitrogen atoms that constitute the chelation cavity, compared to the analogous atoms in the hydrazone derived from pyridoxal-5-phosphate and pyrazine-2-carbohydrazide [28]. This enhanced electron density enables a stronger electrostatic attraction for positively charged metal cations. The observed effect is likely attributable to the pyrazine moiety, which exhibits a stronger electron-withdrawing character than the furan group, thereby polarizing the donor atoms within the cavity.

**Figure 15:**
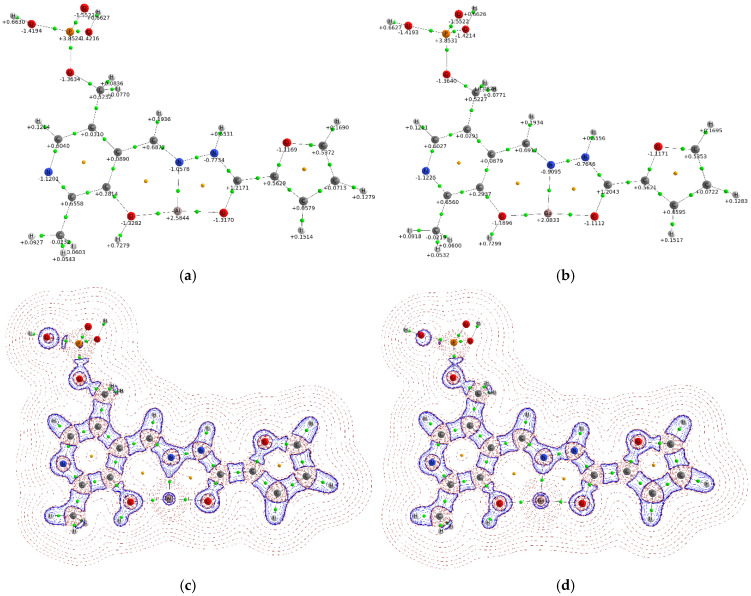
Molecular graph with atomic charges for **1**-Al^3+^ (**a**) and **1**-Ga^3+^ (**b**). The bond critical points are green, and the ring critical points are yellow. The Laplacian map in the plane of coordination cavity O…N…O for the **1**-Al^3+^ complex (**c**) and **1**-Ga^3+^ complex (**d**). The solid lines (blue) correspond to local charge accumulation (∇^2^ρ_b_ < 0) and dashed lines (red) represent a local charge depletion (∇^2^ρ_b_ > 0). The bond critical points are green, and the ring critical point is yellow.

The complexation of probe **1** with aluminum(III) and gallium(III) ions results in a significant redistribution of atomic charges, as shown in Figure 14a and Figure 15a,b. Analysis reveals distinct charge transfer behaviors for the two complexes. In the aluminum(III) complex, the coordinating oxygen and nitrogen atoms acquire a more negative charge compared to the free ligand. In contrast, for the gallium(III) complex, the negative charge on the oxygen atoms diminishes while it increases on the coordinating nitrogen atom. Complexation with Al^3+^ and Ga^3+^ ions does not affect the charge distribution in the phosphate group. The calculated natural charges on the Al^3+^ and Ga^3+^ ions are +2.58e and +2.08e, respectively. These values are lower than the formal +3e charge, indicating a degree of covalent character in the metal-ligand bonds. The greater charge reduction observed for gallium(III) suggests an increase in covalency compared to aluminum(III). This trend can be attributed to the larger ionic radius and lower electronegativity of gallium, which favor enhanced orbital overlap and charge sharing with the donor atoms. Consistent with this, the MEP shows that the region of greatest positive potential in the complexes is localized on the metal ions (Figure S12), contrasting with the distribution in the free ligand. To further elucidate the bonding nature, the electron density distribution was analyzed via Laplacian maps (Figure 15c,d). These maps visualize zones of charge concentration and depletion. The analysis indicates a predominantly ionic interaction, evidenced by significant charge depletion in the internuclear region between the metal ions and the donor atoms of probe **1**. However, notable differences exist between the two metal ions. The aluminum complex exhibits a more pronounced polarization of the lone electron pairs on the donor atoms towards the metal center. This is consistent with the stronger electrostatic field generated by the smaller, more highly charged Al^3+^ ion compared to Ga^3+^.

## 4. Conclusions

In this study, a fluorescent probe based on a vitamin B_6_ derived hydrazone, **1**, was successfully synthesized and characterized. The probe demonstrated high selectivity and sensitivity for the detection of Al^3+^ and Ga^3+^ ions in a buffered aqueous DMSO solution. The recognition mechanism was elucidated through a combination of spectroscopic methods (UV-Vis, fluorescence, and NMR) and density functional theory (DFT) calculations. The probe exhibited a significant “turn-on” fluorescence response upon coordination with Al^3+^ and Ga^3+^, with emission maxima at 466 nm and 477 nm, respectively, and substantial increases in quantum yield. The 1:1 binding stoichiometry was confirmed, with stability constants of log *β′* = 5.98 for **1**-Al^3+^ and log *β′* = 6.28 for **1**-Ga^3+^, indicating a strong affinity, particularly for Ga^3+^. The probe displayed good selectivity for these trivalent cations over a wide range of competing metal ions and anions, with detection limits as low as 48 nM for Al^3+^ and 33 nM for Ga^3+^. DFT calculations provided detailed insights into the molecular structures, electronic transitions, and bonding characteristics of the probe and its metal complexes. The theoretical calculations corroborated the experimental findings, revealing a tridentate coordination mode for both metals via the phenolic oxygen, azomethine nitrogen, and carbonyl oxygen atoms. A notable difference in electronic structure was identified: while the **1**-Al^3+^ complex exhibits ligand-centered transitions, the **1**-Ga^3+^ complex shows a distinct ligand-to-metal charge transfer (LMCT) character, attributed to the accessible d-orbitals of Ga^3+^. Overall, the vitamin B_6_-based hydrazone probe **1** offers a simple, cost-effective, and highly sensitive platform for the selective fluorimetric detection of Al^3+^ and Ga^3+^ ions.

## Figures and Tables

**Table 1:** Key internuclear distances (Å) of probe **1** and its Al^3+^ and Ga^3+^ complexes.

Compound	C-O	C=N	N-N	N-C	C=O	M-O_phen_	M-N	M-O_carb_
1	1.341	1.274	1.347	1.368	1.216	-	-	-
1–Al^3+^	1.453	1.287	1.368	1.345	1.315	1.760	1.841	1.717
1–Ga^3+^	1.447	1.285	1.361	1.346	1.317	1.781	1.886	1.785

## Data Availability

The original contributions presented in the study are included in the article/Supplementary Material; further inquiries can be directed to the corresponding author.

## Supplementary Materials

The following supporting information can be downloaded at: https://www.mdpi.com/article/10.3390/s26092816/s1, Figure S1: IR spectra of probe **1**; Figure S2: MALDI TOF mass spectrum of probe **1**; Figure S3: ^1^H spectra of probe **1**; Figure S4: ^13^C NMR spectra of probe **1**; Figure S5: ^31^P NMR spectra of probe **1**; Figure S6: An example of the results of titration of probe **1** with Al^3+^ ions in the KEV software (stoichiometric model M:L = 1:1) (a), (stoichiometric model M:L = 1:2) (b); Figure S7: An example of the results of titration of probe **1** with Ga^3+^ ions in the KEV software (stoichiometric model M:L = 1:1) (a), (stoichiometric model M:L = 1:2) (b); Figure S8: Calculated UV-Vis spectra of probe **1** and 1-Al^3+^ (stoichiometric model M:L = 1:1) (a), (stoichiometric model M:L = 1:2) (b); Figure S9: Calculated UV-Vis spectra of probe **1** and 1-Ga^3+^ (stoichiometric model M:L = 1:1) (a), (stoichiometric model M:L = 1:2) (b); Table S1: Structures of selected fluorescent probes for Al^3+^ ions in the literature; Table S2: Structures of selected fluorescent probes for Ga^3+^ ions in the literature; Figure S10: The influence of response time on the fluorescence intensity of the probe **1** + Al^3+^ (1 eq.) in DMSO/Tris-HCl, pH 7.4 (9:1 *v*:*v*); Figure S11: Molecular structure of **1**-Al^3+^ (a) and **1**-Ga^3+^ (b); Table S3: Calculated composition of lowest excited states and corresponding oscillator strengths for probe **1**; Table S4: Shapes of molecular orbitals participating in electronic transitions in probe **1**; Table S5: Calculated composition of the lowest excited states and corresponding oscillator strengths for probe **1**-Al^3+^; Table S6: Shapes of molecular orbitals participating in electronic transitions in **1**-Al^3+^; Table S7: Calculated composition of the lowest excited states and corresponding oscillator strengths for probe **1**-Ga^3+^; Table S8: Shapes of molecular orbitals participating in electronic transitions in **1**-Ga^3+^; Figure S12: The molecular electrostatic potential (MEP) mapped on the isodensity surface (0.04 a.u.) for **1**+Al^3+^ (a) and **1**+Ga^3+^ (b).

## Abbreviations

The following abbreviations are used in this manuscript:

AAS	atomic absorption spectroscopy
CAM-B3LYP	Coulomb-Attenuating Method—Becke, 3-parameter, Lee-Yang-Parr
DMSO	dimethyl sulfoxide
DFT	density functional theory
GIAO	Gauge-Including Atomic Orbitals
ICP-MS	inductively coupled plasma mass spectrometry
MALDI TOF	Matrix-assisted laser desorption/ionization
UV-Vis	Ultraviolet–visible spectroscopy
