# Supplementary material for: Experimental and DFT Investigation of a Vitamin B6-Derived Fluorescent Probe for Detection of Al3+ and Ga3+ Ions in a Buffered Aqueous DMSO Solution
Source: Sensors (Basel). 2026 Apr 30;26(9):2816. doi: 10.3390/s26092816 (PMC13165742; doi:10.3390/s26092816)
Supplement: Supplementary file 1 [file sensors-26-02816-s001.zip › sensors-4268355-supplementary.pdf]

Supporting Information

Experimental and DFT Investigation of a Vitamin B_6-Derived Fluorescent Probe for Detection of Al^{3+} and Ga^{3+} Ions in a Buffered Aqueous DMSO Solution

Maksim N. Zavalishin *, Artemiy A. Guschin and George A. Gamov

General Chemical Technology Department, Ivanovo State University of Chemistry and Technology, Sheremetevskii Ave. 7, 153000 Ivanovo, Russia;
artemiygushiin@gmail.com (A.A.G.); ggamov@isuct.ru (G.A.G.)
* Correspondence: zavalishin00@gmail.com; Tel.: +7-9106757233

Table of Contents

1. **Figure S1** – IR spectra of probe **1**
2. **Figure S2** – MALDI TOF mass spectrum of probe **1**
3. **Figure S3** – ^{1}H NMR spectra of probe **1**
4. **Figure S4** – ^{13}C NMR spectra of probe **1**
5. **Figure S5**– ^{31}P NMR spectra of probe **1**
6. **Figure S6** – An example of the results of titration of probe **1** with Al^{3+} ions in the KEV software (stoichiometric model M:L = 1:1) (a), (stoichiometric model M:L = 1:2) (b)
7. **Figure S7** – An example of the results of titration of probe **1** with Ga^{3+} ions in the KEV software (stoichiometric model M:L = 1:1) (a), (stoichiometric model M:L = 1:2) (b)
8. **Figure S8** – Calculated UV-Vis spectra of probe **1** and **1**-Al^{3+} (stoichiometric model M:L = 1:1) (a), (stoichiometric model M:L = 1:2) (b)
9. **Figure S9** – Calculated UV-Vis spectra of probe **1** and **1**-Ga^{3+} (stoichiometric model M:L = 1:1) (a), (stoichiometric model M:L = 1:2) (b)
10. **Table S1**. Structures of selected fluorescent probes for Al^{3+} ions in the literature
11. **Table S2**. Structures of selected fluorescent probes for Ga^{3+} ions in the literature
12. **Figure S10** – The influence of response time on the fluorescence intensity of the probe **1** + Al^{3+} (1 eq.) in DMSO/Tris-HCl, pH 7.4 (9:1 v:v)
13. **Figure S11** –Molecular structure of **1**-Al^{3+} (a) and **1**-Ga^{3+} (b)
14. **Table S3.** Calculated composition of lowest excited states and corresponding oscillator strengths for probe **1**
15. **Table S4.** Shapes of molecular orbitals participating in electronic transitions in probe **1**
16. **Table S5**. Calculated composition of the lowest excited states and corresponding oscillator strengths for probe **1**-Al^{3+}
17. **Table S6.** Shapes of molecular orbitals participating in electronic transitions in **1**-Al^{3+}
18. **Table S5**. Calculated composition of the lowest excited states and corresponding oscillator strengths for probe **1**-Ga^{3+}
19. **Table S6.** Shapes of molecular orbitals participating in electronic transitions in **1**-Ga^{3+}
20. **Figure S12.** The molecular electrostatic potential (MEP) mapped on the isodensity surface (0.04 a.u.) for **1**+Al^{3+} (a) and **1**+Ga^{3+} (b)

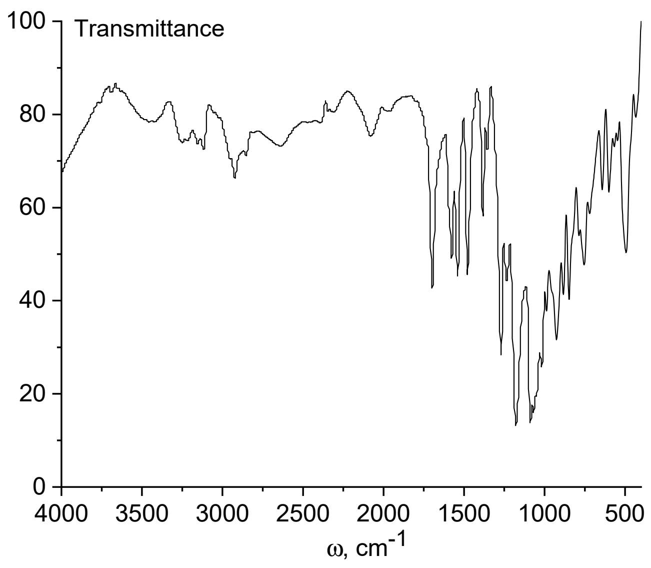

Figure S1 – IR spectra of probe **1**

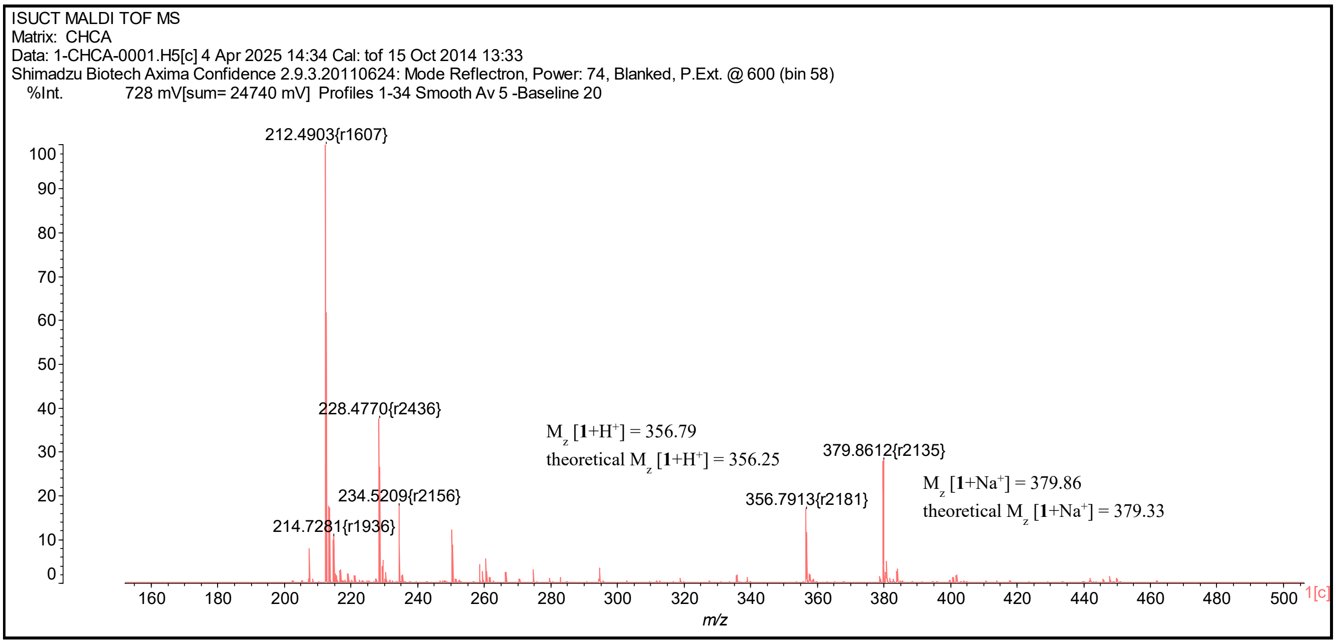

Figure S2 – MALDI TOF mass spectrum of probe **1**

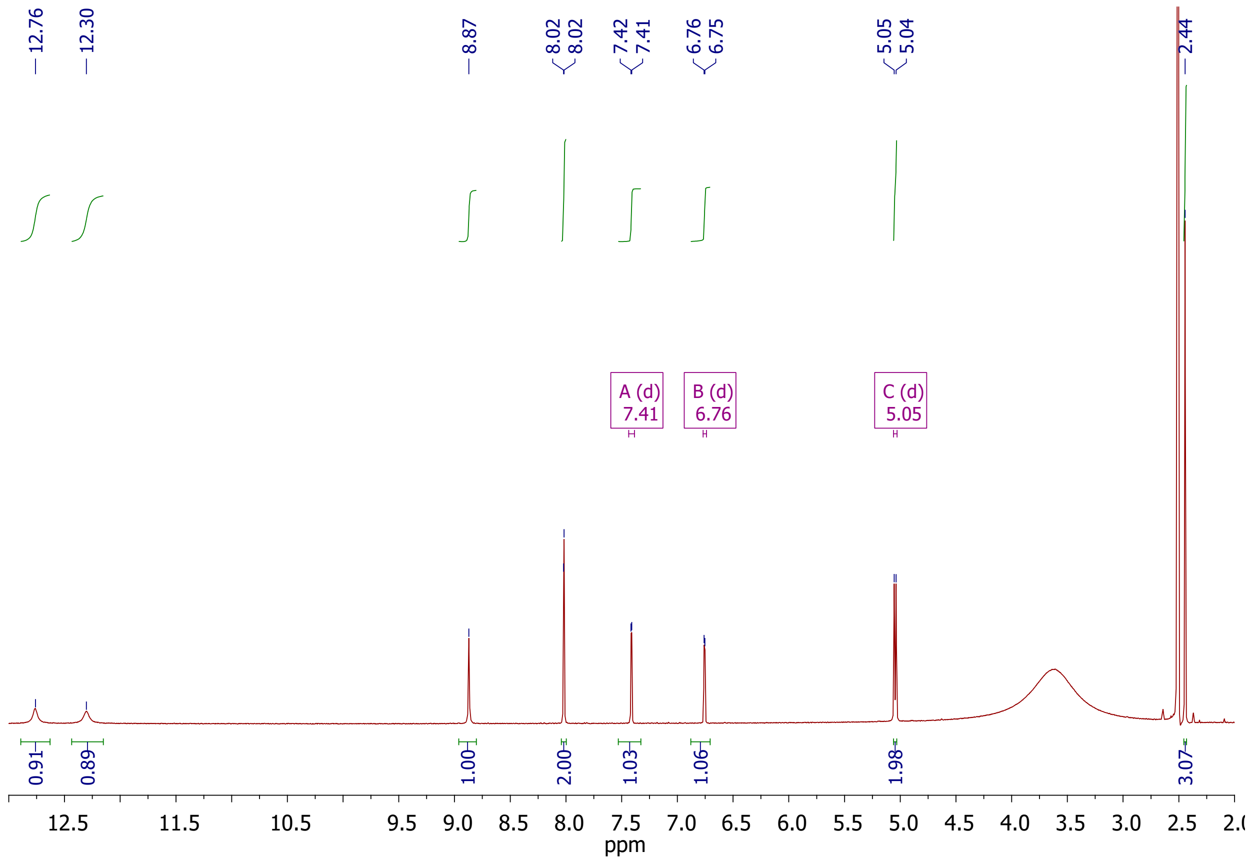

Figure S3 – ^{1}H NMR spectra of probe **1**

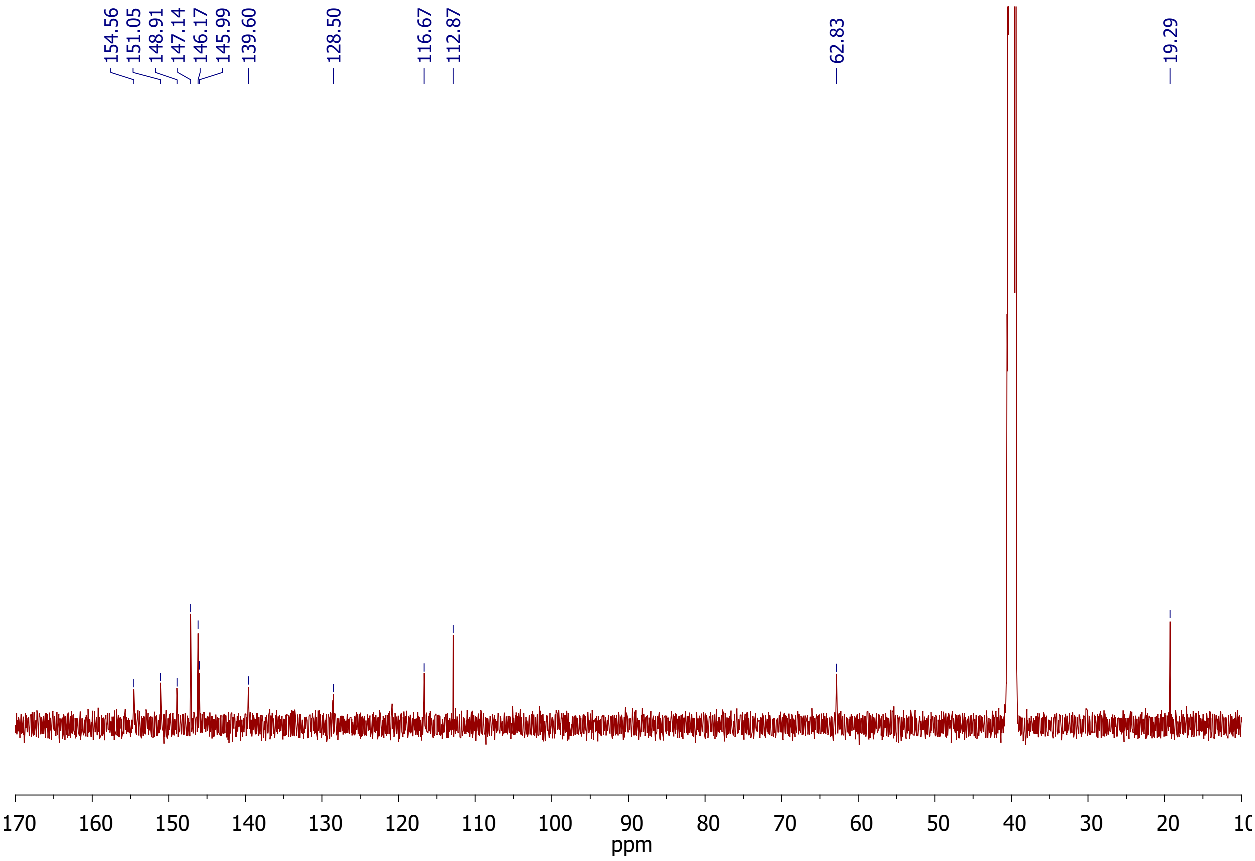

Figure S4 – ^{13}C NMR spectra of probe **1**

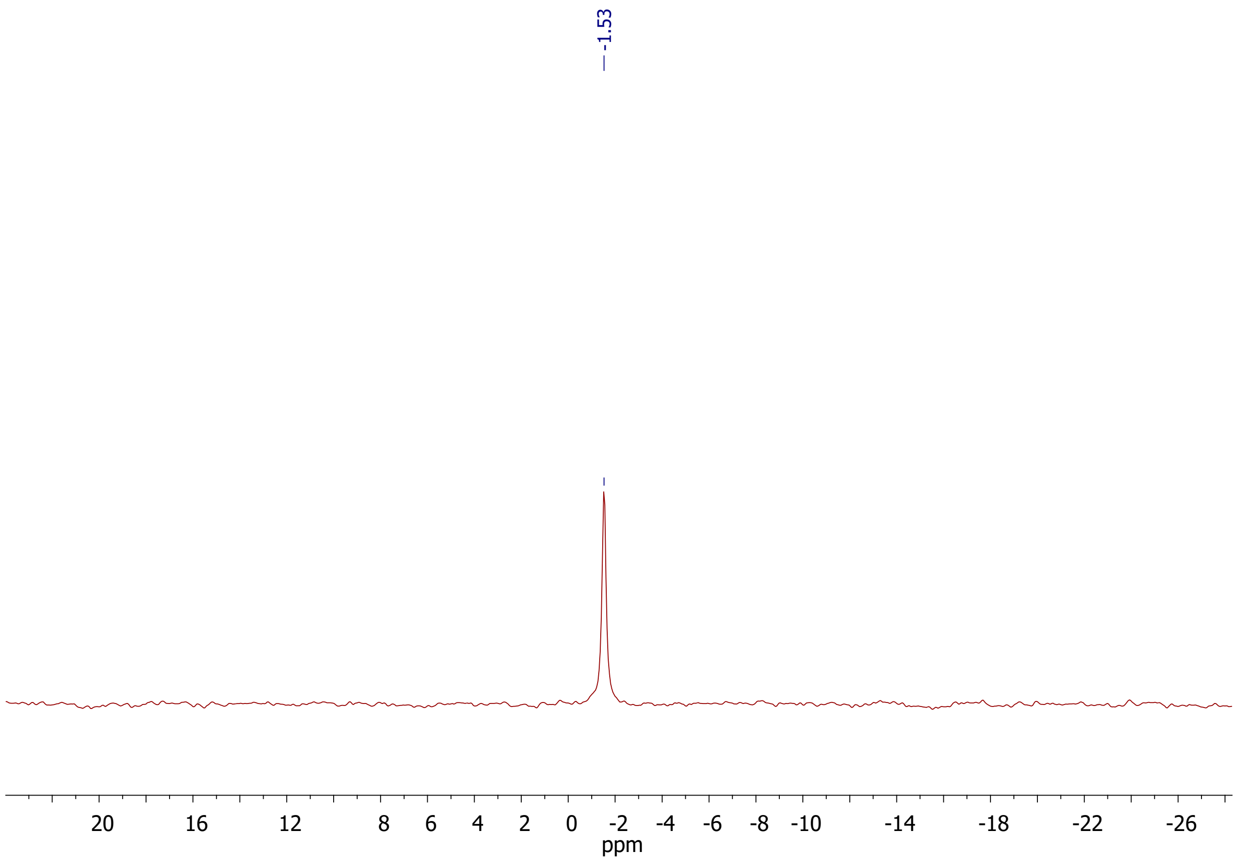

Figure S5 – ^{31}P NMR spectra of probe **1**

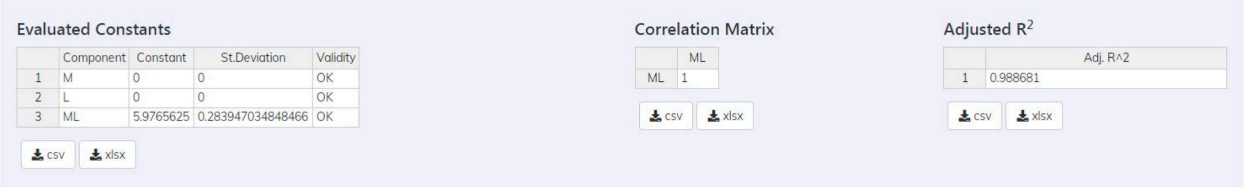

Evaluated Constants

	Component	Constant	St.Deviation	Validity
1	M	0	0	OK
2	L	0	0	OK
3	ML	5.9765625	0.283947034848466	OK

csv xlsx

Correlation Matrix

	ML
ML	1

csv xlsx

Adjusted R^2

	Adj. R^2
1	0.988681

csv xlsx

(a)

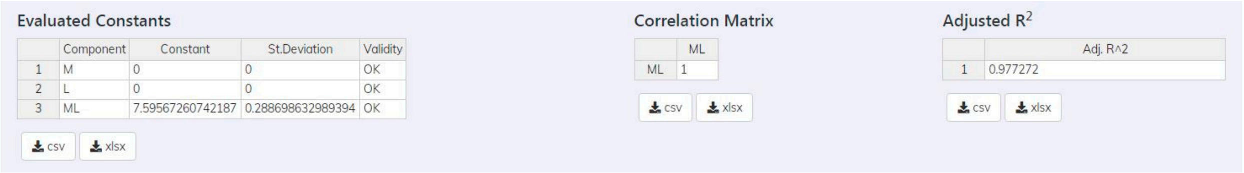

Evaluated Constants

	Component	Constant	St.Deviation	Validity
1	M	0	0	OK
2	L	0	0	OK
3	ML	7.59567260742187	0.288698632989394	OK

csv xlsx

Correlation Matrix

	ML
ML	1

csv xlsx

Adjusted R^2

	Adj. R^2
1	0.977272

csv xlsx

(b)

Figure S6 – An example of the results of titration of probe **1** with Al^{3+} ions in the KEV software (stoichiometric model M:L = 1:1) (a), (stoichiometric model M:L = 1:2) (b)

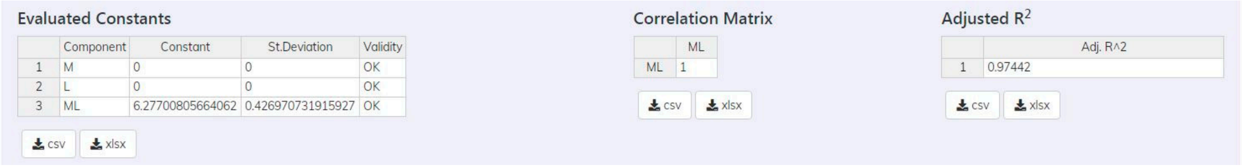

Evaluated Constants

	Component	Constant	St.Deviation	Validity
1	M	0	0	OK
2	L	0	0	OK
3	ML	6.27700805664062	0.426970731915927	OK

csv xlsx

Correlation Matrix

	ML
ML	1

csv xlsx

Adjusted R^2

	Adj. R^2
1	0.97442

csv xlsx

(a)

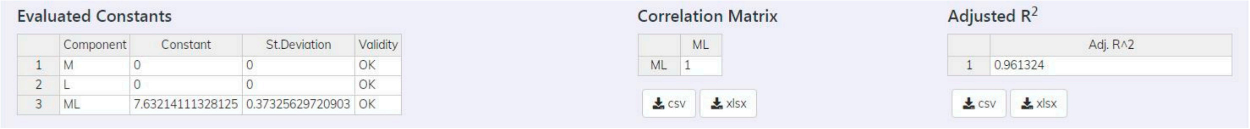

Evaluated Constants

	Component	Constant	St.Deviation	Validity
1	M	0	0	OK
2	L	0	0	OK
3	ML	7.63214111328125	0.37325629720903	OK

Correlation Matrix

	ML
ML	1

csv xlsx

Adjusted R^2

	Adj. R^2
1	0.961324

csv xlsx

(b)

Figure S7 – An example of the results of titration of probe **1** with Ga^{3+} ions in the KEV software (stoichiometric model M:L = 1:1) (a), (stoichiometric model M:L = 1:2) (b)

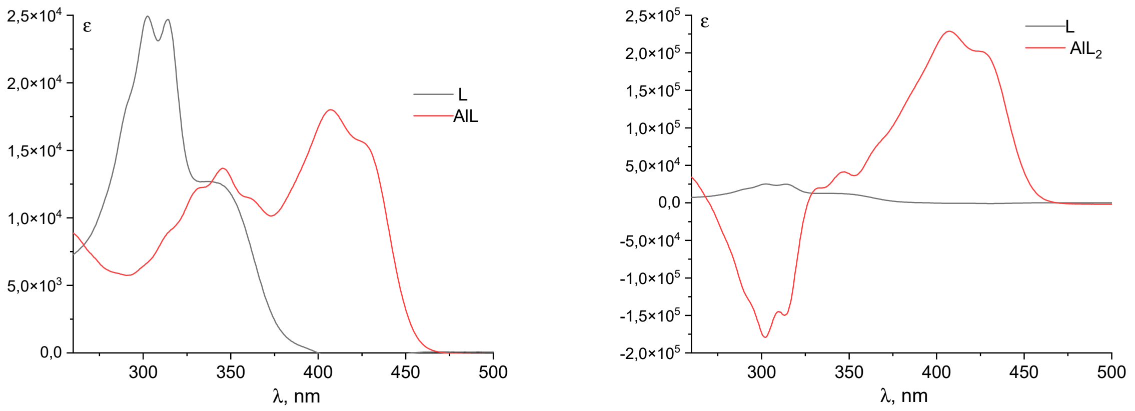

(a) (b)

Figure S8 – Calculated UV-Vis spectra of probe **1** and **1**-Al^{3+} (stoichiometric model M:L = 1:1) (a), (stoichiometric model M:L = 1:2) (b)

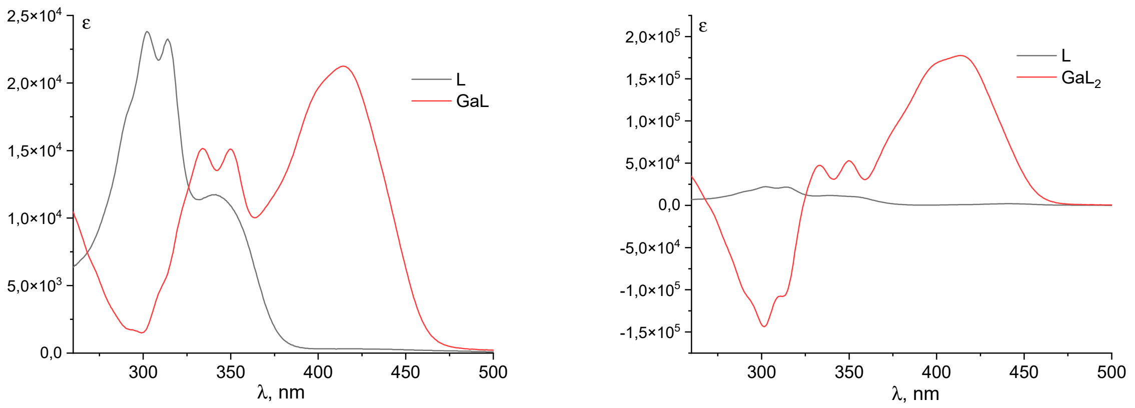

(a) (b)

Figure S9 – Calculated UV-Vis spectra of probe **1** and **1**-Ga^{3+} (stoichiometric model M:L = 1:1) (a), (stoichiometric model M:L = 1:2) (b)

Table S1. Structures of selected fluorescent probes for Al^{3+} ions in the literature

Ref.	Year	Structure	Signal type	$\lambda_{ex}/\lambda_{em}$, nm	LOD (μM)	Solvent	Application
[1]	2018	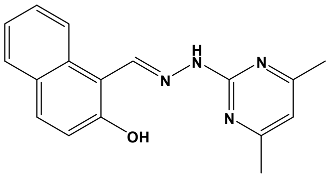	turn-on	380/ 470	2.78	EtOH	logic gate, cells
[2]	2015	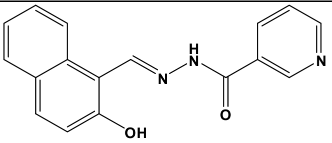	turn-on	409/ 474	4.0	Tris–HCl, pH 7.2	-
[3]	2016	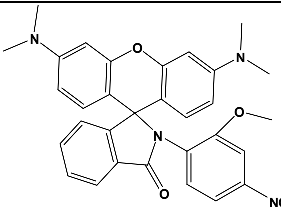	turn-on	520/ 580	0.32	$EtOH/H_2O$ (2:3, v/v)	cells
[4]	2022	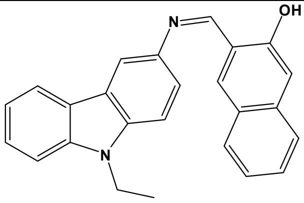	turn-on	320/ 533	0.26	$EtOH/H_2O$ (1:1, v/v)	-
[5]	2020	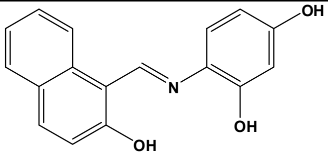	turn-on	385	0.49	DMF/H_2O (9:1, v/v)	Test strips
[6]	2021	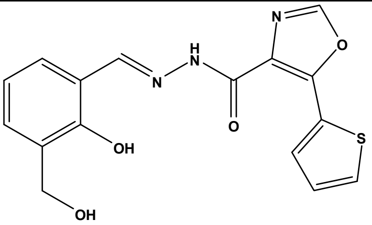	turn-on	365/ 476	0.55	DMF–H2O (4:6, v/v)	Water, food, cells
This work	2026	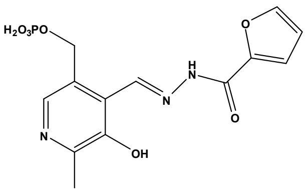	turn-on	411/ 466	0.048	DMSO/ H_2O (9:1 v:v)	-

References:

[1] B. Das, S. Dey, G.P. Maiti, A. Bhattacharjee, A. Dhara, A. Jana, Hydrazinopyrimidine derived novel Al3+ chemosensor: molecular logic gate and biological applications, New J. Chem. 42 (2018) 9424–9435. https://doi.org/10.1039/C7NJ05095J.

[2] J. Qin, Z. Yang, P. Yang, Recognition of Al3+ based on a naphthalene-based “Off–On” chemosensor in near 100% aqueous media, Inorganica Chim. Acta 432 (2015) 136–141. https://doi.org/10.1016/j.ica.2015.03.029.

[3] N. Chatterjee, S.B. Maity, A. Samadder, P. Mukherjee, A.R. Khuda-Bukhsh, P.K. Bharadwaj, A chemosensor for Al3+ ions in aqueous ethanol media: photophysical and live cell imaging studies, RSC Adv. 6 (2016) 17995–18001. https://doi.org/10.1039/C5RA23968K.

[4] F. Kolcu, İ. Kaya, Carbazole-based Schiff base: A sensitive fluorescent ‘turn-on’ chemosensor for recognition of Al(III) ions in aqueous-alcohol media, Arab. J. Chem. 15 (2022) 103935. https://doi.org/10.1016/j.arabjc.2022.103935.

[5] A. Mondal, E. Ahmmed, S. Chakraborty, A. Sarkar, S. Lohar, P. Chattopadhyay, Aggregation Induced Emission Enhancement (AIEE) of Naphthalene- Appended Organic Moiety: An Al3+ Ion Selective Turn-On Fluorescent Probe, ChemistrySelect 5 (2020) 147–155. https://doi.org/10.1002/slct.201903645.

[6] H. Chen, P. Luo, B. Hu, X. Cheng, Y. Yang, F. Yan, X. Lyu, Q. Xing, D. Zhou, Thiophene-oxazolyl hydrazone-functionalized chemosensor for ultrasensitive detection of Al3+ in water, hydrogel, food, and living cells, Spectrochim. Acta. A. Mol. Biomol. Spectrosc. 349 (2026) 127389. https://doi.org/10.1016/j.saa.2025.127389.

[7] X. Chen, W. Qin, Y. Zhao, Y. Qin, F.-M. Long, B. Chen, Z. Xing, A facile fluorescence probe for the recognition and distinguishment of Al3+ and Ga3+ and its application, Inorganica Chim. Acta 583 (2025) 122707. https://doi.org/10.1016/j.ica.2025.122707.

[8] L. Yan, S. Zhang, Y. Xie, C. Lei, A fluorescent probe for Gallium(Ⅲ) ions based on 2-hydroxy-1-naphthaldehyde and L-serine, Dyes Pigments 175 (2020) 108190. https://doi.org/10.1016/j.dyepig.2020.108190.

[9] X. He, C. Wu, Y. Qian, Y. Li, F. Ding, Z. Zhou, J. Shen, Symmetrical bis-salophen probe serves as a selectively and sensitively fluorescent switch of gallium ions in living cells and zebrafish, Talanta 205 (2019) 120118. https://doi.org/10.1016/j.talanta.2019.120118.

[10] J.Y. Noh, S. Kim, I.H. Hwang, G.Y. Lee, J. Kang, S.H. Kim, J. Min, S. Park, C. Kim, J. Kim, Solvent-dependent selective fluorescence assay of aluminum and gallium ions using julolidine-based probe, Dyes Pigments 99 (2013) 1016–1021. https://doi.org/10.1016/j.dyepig.2013.07.035.

[11] Y. Xing, Z. Liu, B. Li, L. Li, X. Yang, G. Zhang, The contrastive study of two thiophene-derived symmetrical Schiff bases as fluorescence sensors for Ga3+ detection, Sens. Actuators B Chem. 347 (2021) 130497. https://doi.org/10.1016/j.snb.2021.130497.

[12] M.N. Zavalishin, M.A. Maltseva, V.S. Osokin, V.V. Aleksandriiskii, U.A. Petrova, A.A. Knyazeva, A.V. Eroshin, Yu.A. Zhabanov, G.A. Gamov, Synthesis and characterization of a vitamin B6-tetrazole hydrazone as a fluorescence probe for selective detection of Cd2+ and Ga3+ ions, Opt. Mater. 158 (2025) 116493. https://doi.org/10.1016/j.optmat.2024.116493.

Table S2. Structures of selected fluorescence probes for Ga^{3+} ions in the literature

Ref.	Year	Structure	Signal type	$\lambda_{ex}/\lambda_{em}$, nm	LOD (μM)	Solvent	Application
[7]	2018	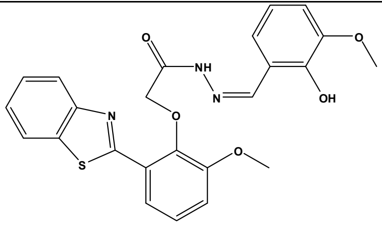	turn-on	400/ 492	0.032	DMF/H_2O (v/v, 1/1)	Water
[8]	2020	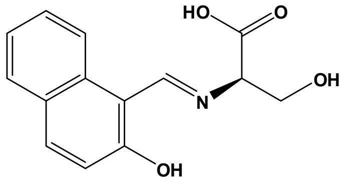	turn-on	380/ 450	0.11	(PBS:DMSO = 9:1, v/v, pH 7.4)	Cells
[9]	2019	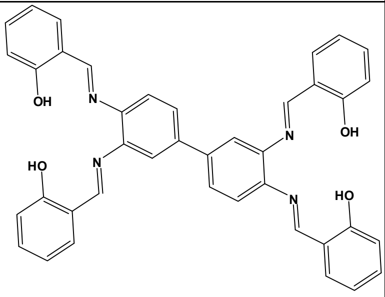	turn-on	350/ 500	0.013	DMSO/ H_2O (1:1)	Cells, zebrafish
[10]	2013	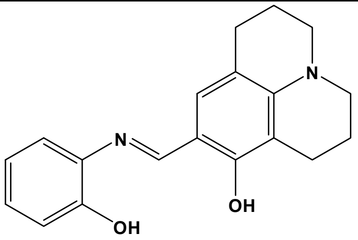	turn-on	450/ 516	0.10	Tris buffer (pH = 7.0)	Cells
[11]	2021	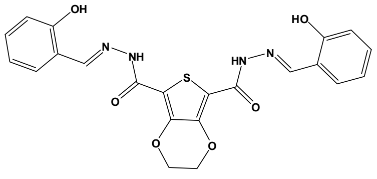	turn-on	421/ 513	0.086	MeCN/ Tris buffer, pH 7.4 (9:1, v/v)	Test strips
[12]	2025	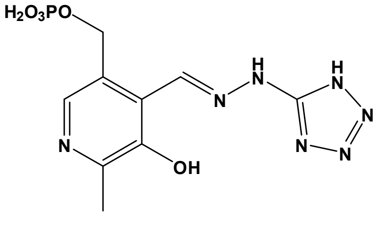	turn-on	380/ 525	0.97	DMSO/ HEPES buffer, pH 7.4 (9/1 v./v.)	-

This work	2026	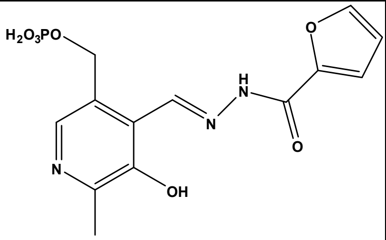	turn-on	411/ 477	0.033	DMSO/Tris-HCl, pH 7.4 (9:1 v:v).	-

Referenses:

[7] D. Yun, J.M. Jung, C. Kim, A fluorescent and colorimetric chemosensor for Ga3+ and CN–, Inorganica Chim. Acta 479 (2018) 154–160. https://doi.org/10.1016/j.ica.2018.04.021.

[8] L. Yan, S. Zhang, Y. Xie, C. Lei, A fluorescent probe for Gallium(Ⅲ) ions based on 2-hydroxy-1-naphthaldehyde and L-serine, Dyes Pigments 175 (2020) 108190. https://doi.org/10.1016/j.dyepig.2020.108190.

[9] X. He, C. Wu, Y. Qian, Y. Li, F. Ding, Z. Zhou, J. Shen, Symmetrical bis-salophen probe serves as a selectively and sensitively fluorescent switch of gallium ions in living cells and zebrafish, Talanta 205 (2019) 120118. https://doi.org/10.1016/j.talanta.2019.120118.

[10] J.Y. Noh, S. Kim, I.H. Hwang, G.Y. Lee, J. Kang, S.H. Kim, J. Min, S. Park, C. Kim, J. Kim, Solvent-dependent selective fluorescence assay of aluminum and gallium ions using julolidine-based probe, Dyes Pigments 99 (2013) 1016–1021. https://doi.org/10.1016/j.dyepig.2013.07.035.

[11] Y. Xing, Z. Liu, B. Li, L. Li, X. Yang, G. Zhang, The contrastive study of two thiophene-derived symmetrical Schiff bases as fluorescence sensors for Ga3+ detection, Sens. Actuators B Chem. 347 (2021) 130497. https://doi.org/10.1016/j.snb.2021.130497.

[12] M.N. Zavalishin, G.A. Gamov, A.E. Pogonin, A.K. Isagulieva, A.V. Shibaeva, M.A. Klimovich, V.N. Morozov, A new fluorescent vitamin B6-based probe for selective and sensitive detection Ga3+ ions in the environment and living cells, Dyes Pigments 219 (2023) 111621. https://doi.org/10.1016/j.dyepig.2023.111621.

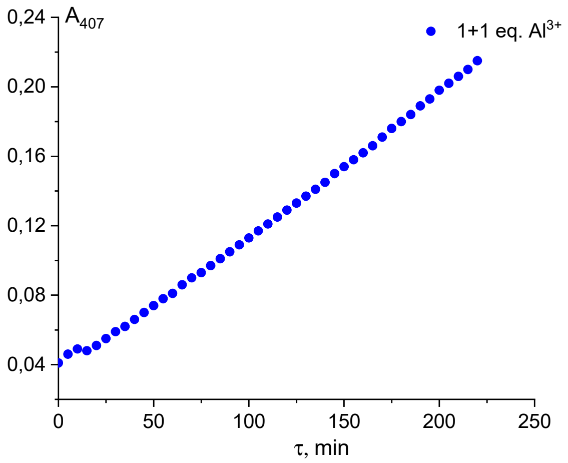

Figure S10 – The influence of response time on the fluorescence intensity of the probe **1** + Al^{3+} (1 eq.) in DMSO/Tris-HCl, pH 7.4 (9:1 v:v)

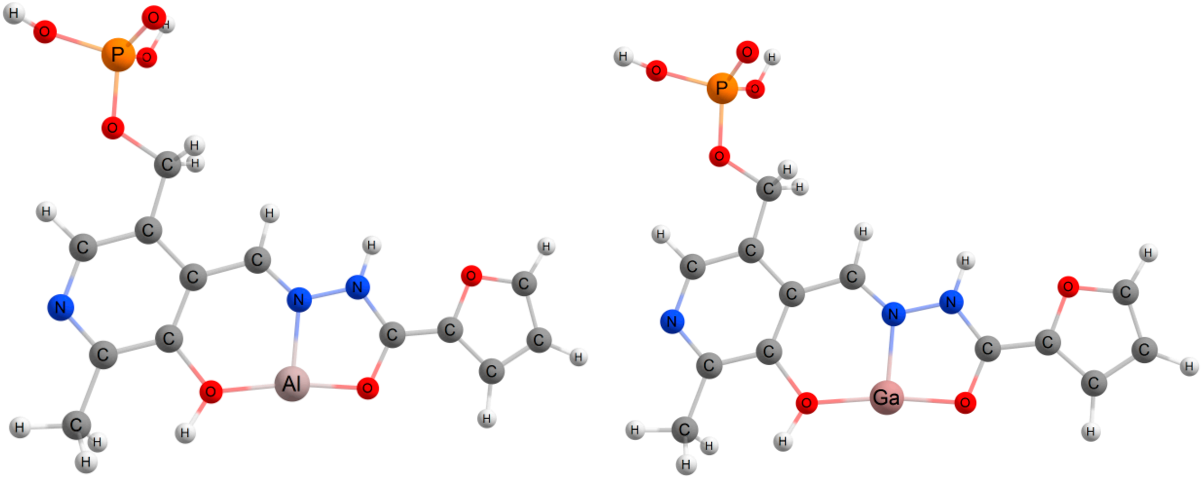

(a) (b)

Figure S11 –Molecular structure of **1**-Al^{3+} (a) and **1**-Ga^{3+} (b)

Table S3. Calculated composition of lowest excited states and corresponding oscillator strengths for prob**e 1**

State	Composition, (%)	λ, nm	λ exp, nm	oscillator strength, f[a]
	HOMO-1→LUMO+1 (2)			
	HOMO→LUMO (95)			
1A	HOMO→LUMO+1 (3)	311		0.4712
	HOMO-2→LUMO (10)			
	HOMO-2→LUMO+1 (4)			
	HOMO-1→LUMO (82)			
2A	HOMO→LUMO+1 (4)	273	304	0.7153
	HOMO-2→LUMO (68)			
	HOMO-2→LUMO+1 (6)			
	HOMO-1→LUMO (4)			
	HOMO-1→LUMO+1 (18)			
5A	HOMO→LUMO+1 (4)	239		0.1427
	HOMO-5→LUMO (4)			
	HOMO-5→LUMO (3)			
	HOMO-2→LUMO (8)			
	HOMO-2→LUMO+1 (5)			
	HOMO-1→LUMO (5)			
	HOMO-1→LUMO+1 (14)			
	HOMO→LUMO+1 (54)			
7A	HOMO→ LUMO+2 (7)	212		0.0543
	HOMO-6→LUMO (11)			
	HOMO-5→LUMO (4)			
	HOMO-1→LUMO (4)			
	HOMO-1→ LUMO+2 (2)			
	HOMO-1→ LUMO+4 (2)			
	HOMO→LUMO+1 (14)			
8A	HOMO→ LUMO+2 (63)	208		0.1747

[a] bands with f>0.05 presented

Table S4. Shapes of molecular orbitals participating in electronic transitions in probe **1**

Orbital	Energy, Ev	View
HOMO-6	-10.08	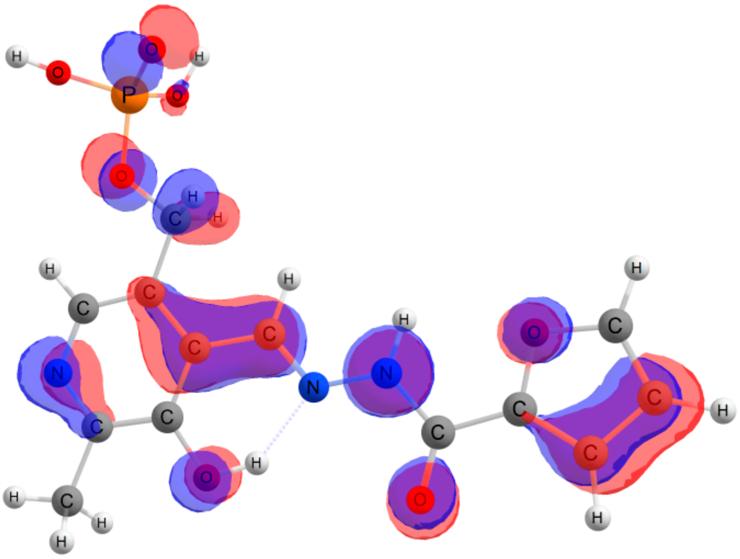
HOMO-5	-9.90	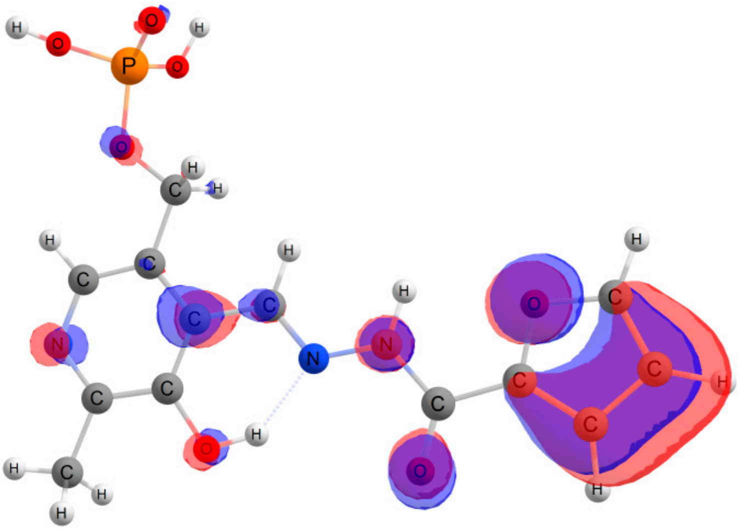
HOMO-4	-9.72	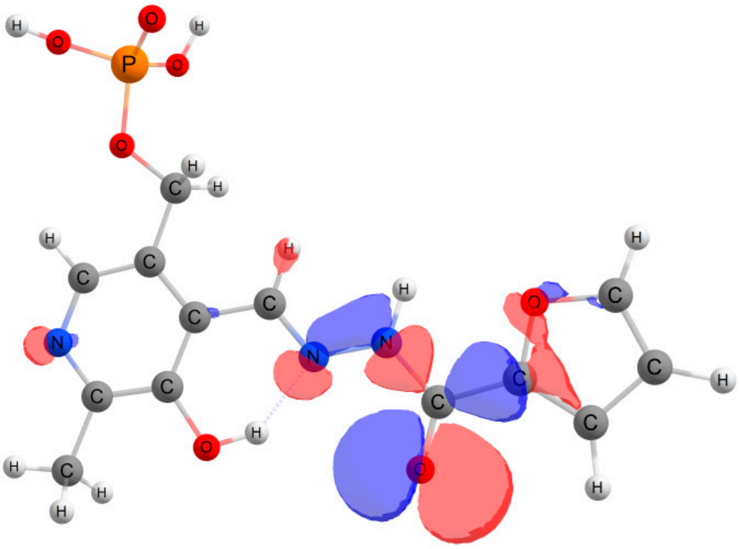

HOMO-3	-8.98	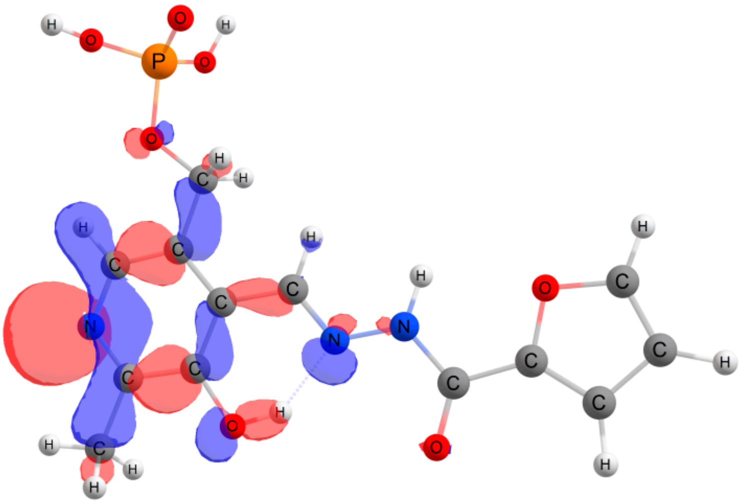
HOMO-2	-8.64	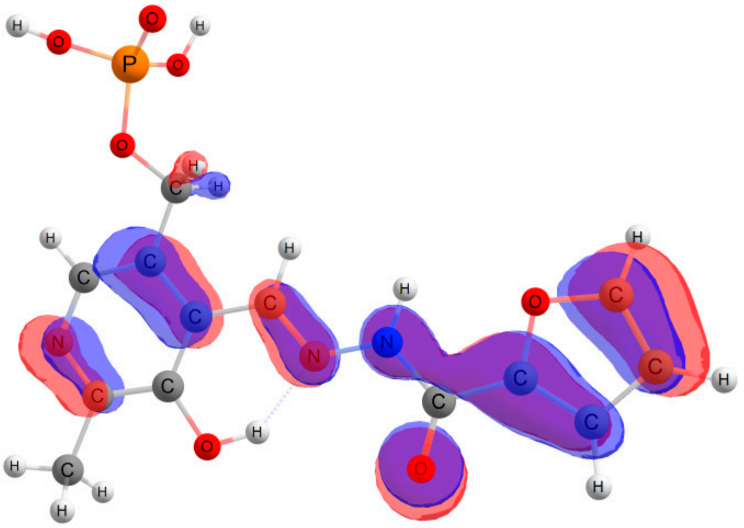
HOMO-1	-8.28	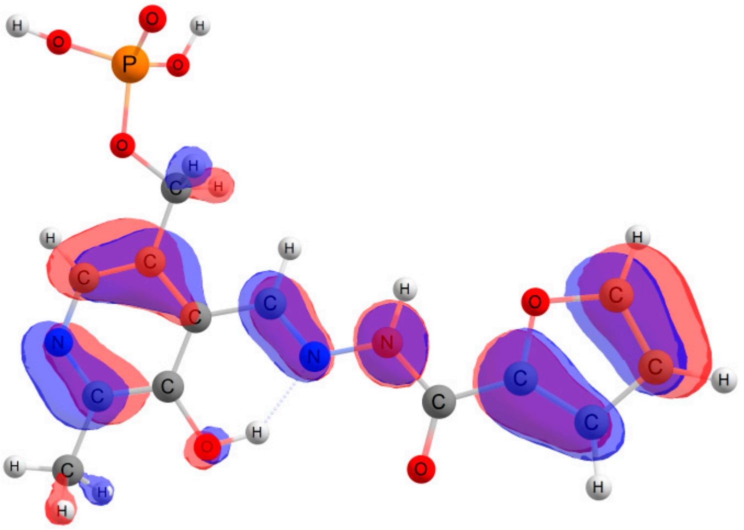

HOMO	-7.79	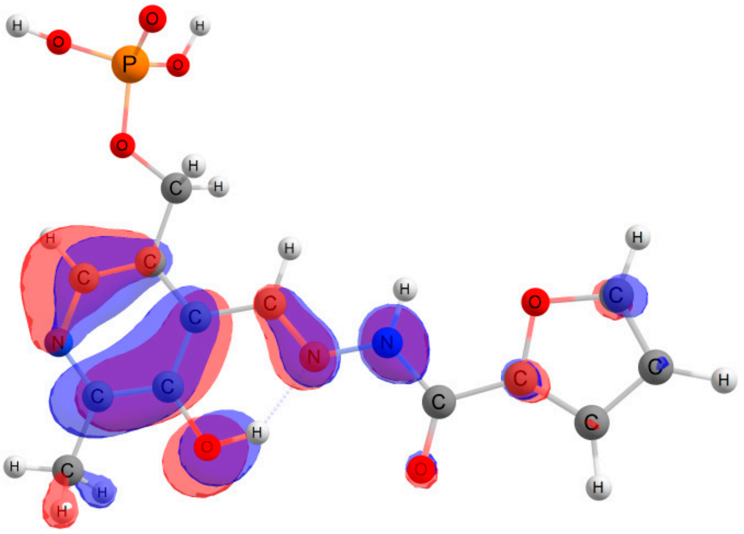
LUMO	-1.12	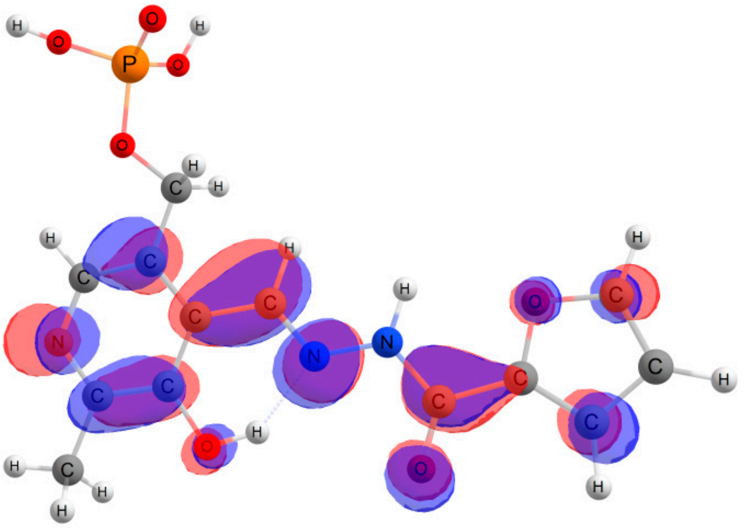
LUMO+1	0.12	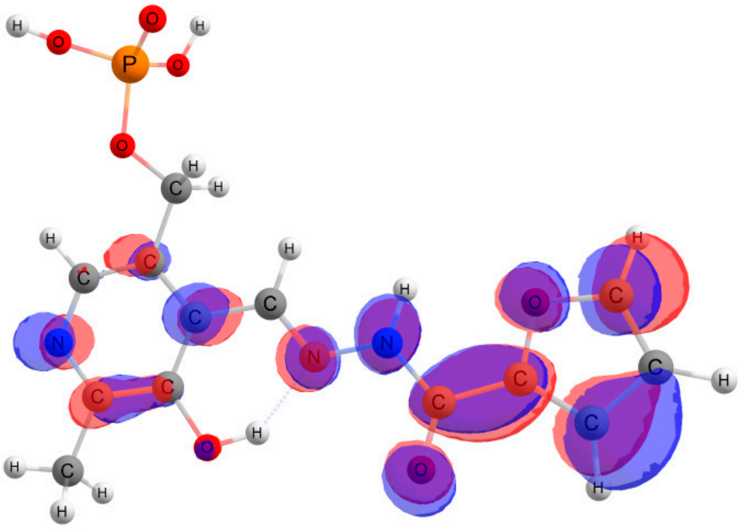

LUMO+2	1.02	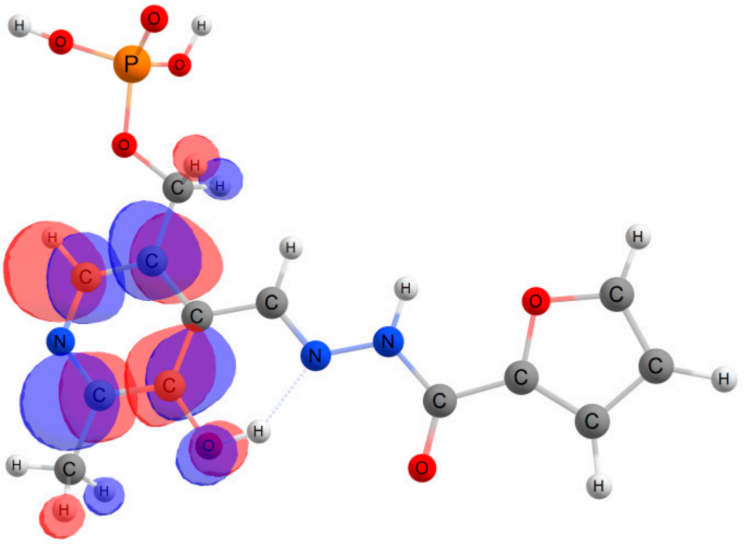
LUMO+3	1.55	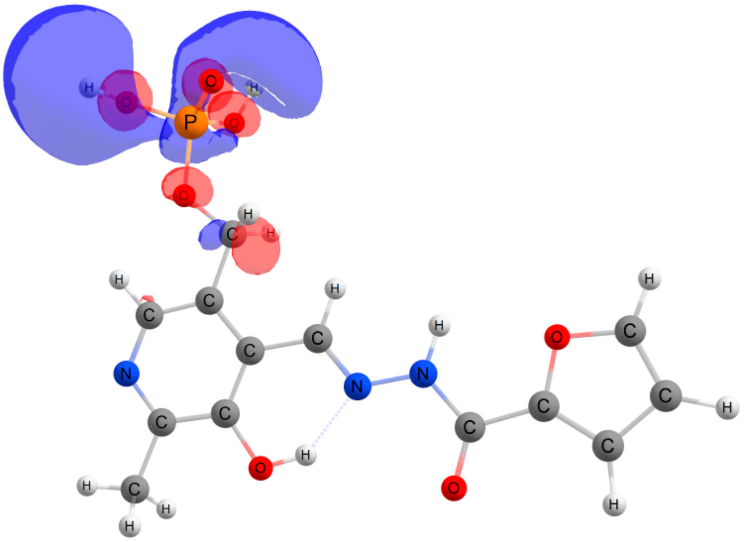
LUMO+4	1.85	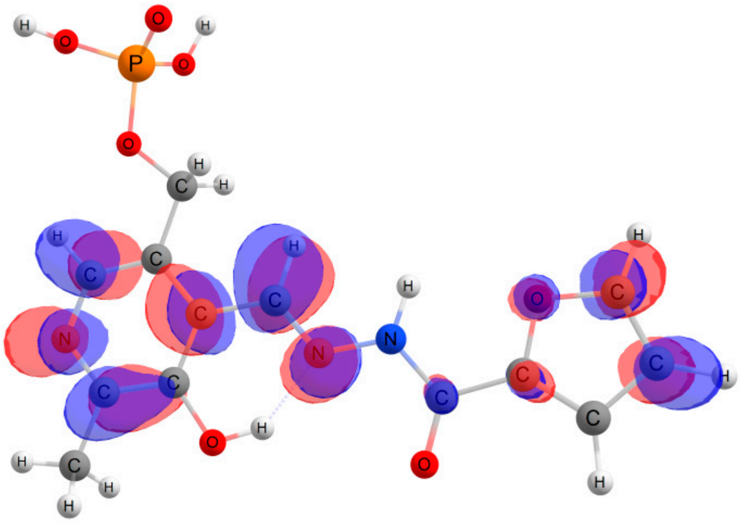

Table S5. Calculated composition of the lowest excited states and corresponding oscillator strengths for probe **1**-Al^{3+}

State	Composition, (%)	λ, nm	λ exp, nm	oscillator strength, f[a]
	HOMO-1→LUMO (24)			
	HOMO-1→LUMO+2 (3)			
1A	HOMO→LUMO (73)	365		0.8691
	HOMO-5→LUMO (2)			
	HOMO-1→LUMO (73)			
	HOMO→LUMO (21)			
3A	HOMO→LUMO+2 (4)	339		0.7153
	HOMO-6→LUMO (68)			
	HOMO-4→LUMO (6)			
	HOMO-3→LUMO (4)			
	HOMO-2→LUMO+1 (18)			
	HOMO→LUMO (4)			
	HOMO→LUMO+2 (4)			
7A	HOMO→LUMO+2 (63)	253		0.0658

[a] bands with f>0.05 presented

Table S6. Shapes of molecular orbitals participating in electronic transitions in **1**-Al^{3+}

Orbital	Energy, Ev	View
HOMO-6	-11.35	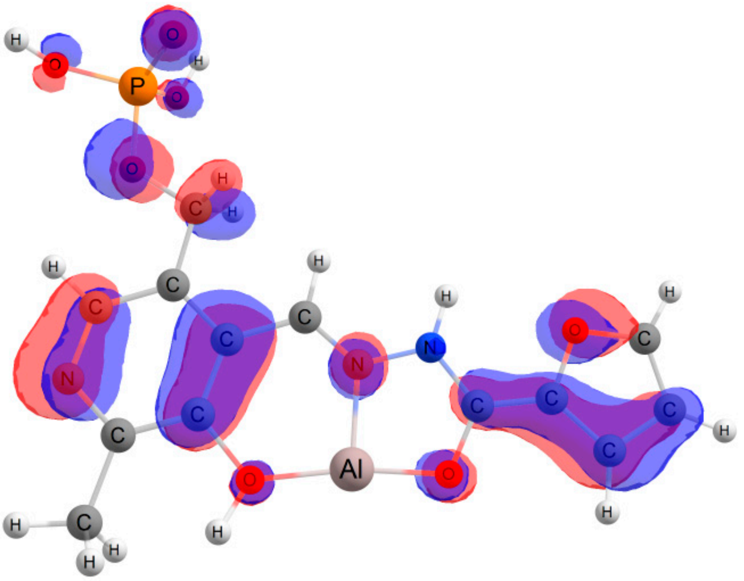
HOMO-5	-11.09	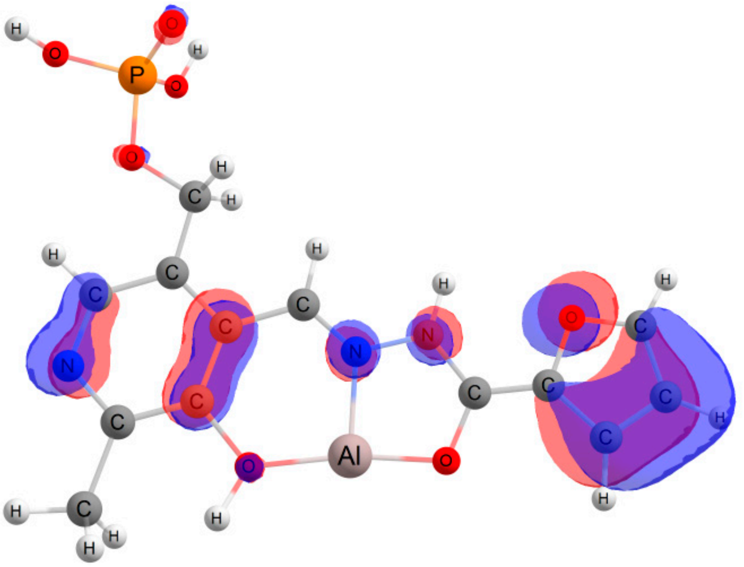

HOMO-4	-10.68	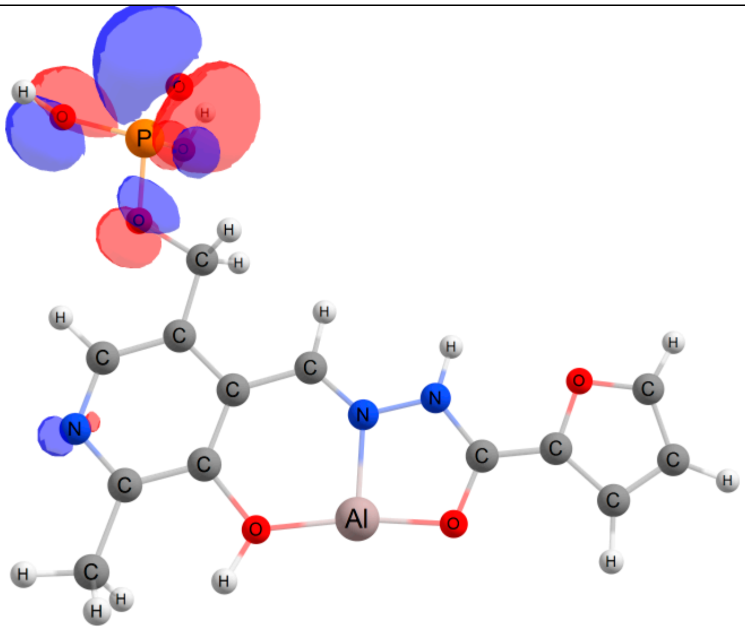
HOMO-3	-10.62	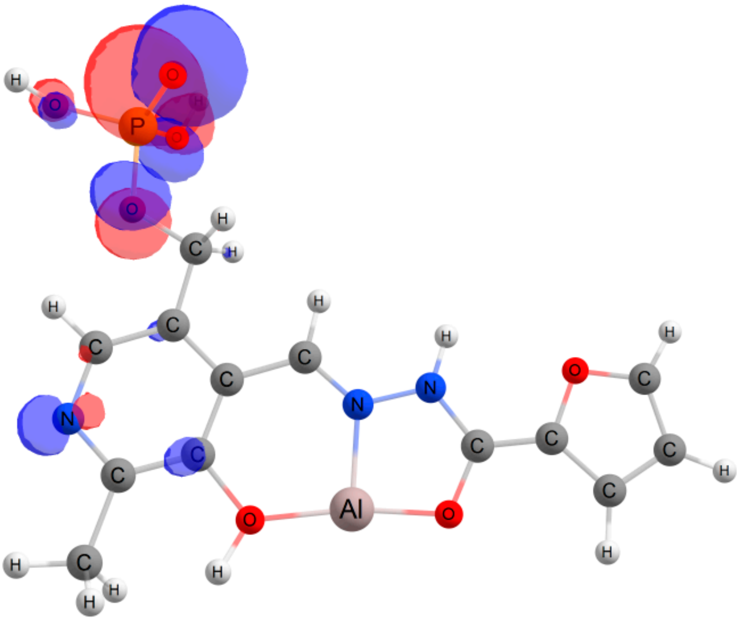
HOMO-2	-10.45	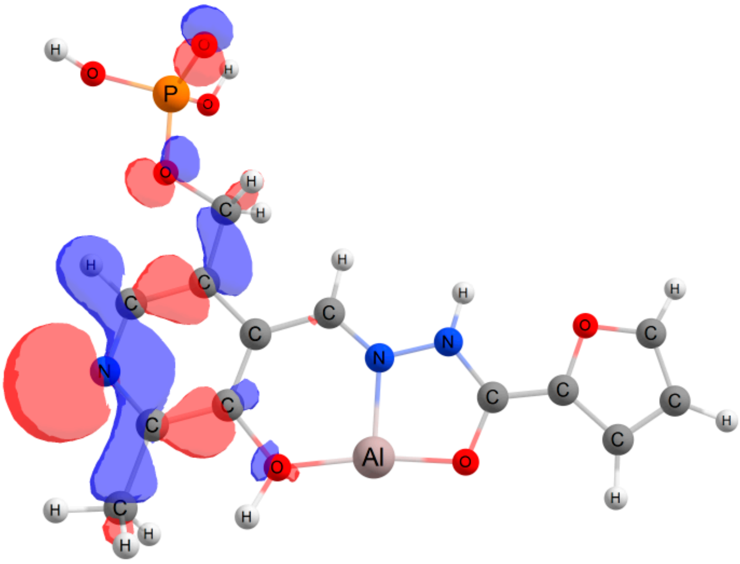

HOMO-1	-10.09	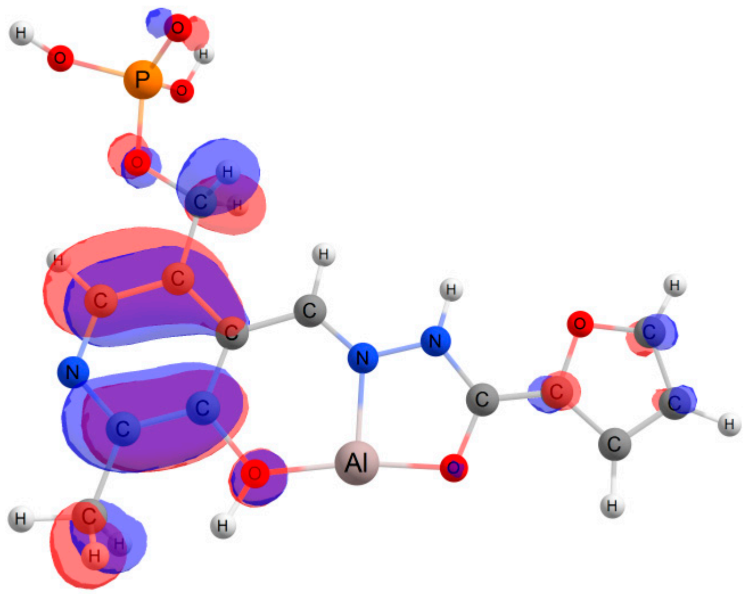
HOMO	-9.63	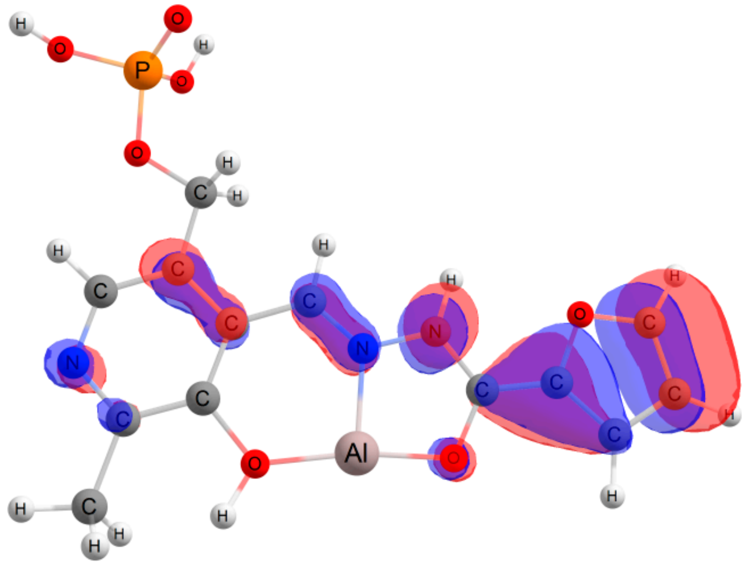
LUMO	-3.95	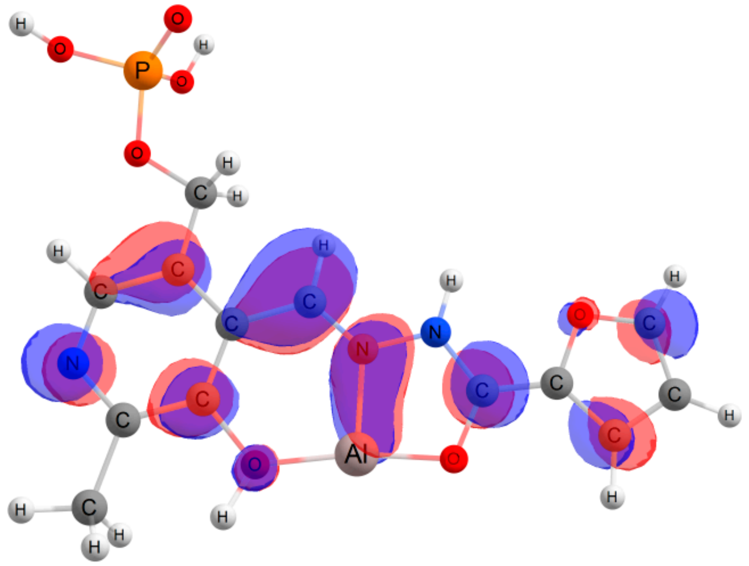

LUMO+ 1	-3.63	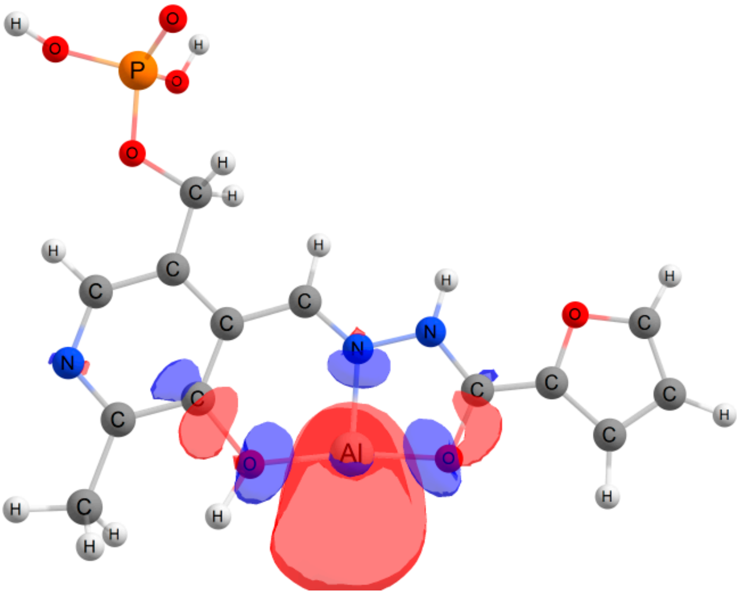
LUMO+ 2	-2.34	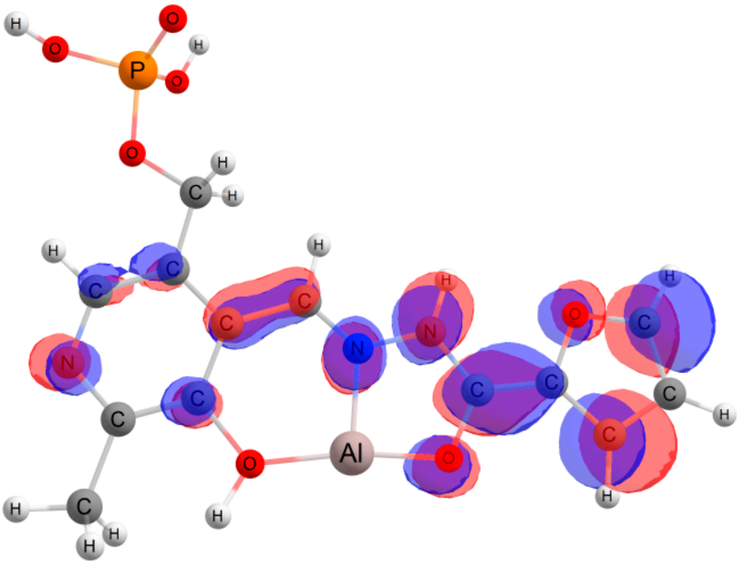

Table S7. Calculated composition of the lowest excited states and corresponding oscillator strengths for probe **1**-Ga^{3+}

State	Composition, (%)	λ, nm	λ exp, nm	oscillator strength, f[a]
2A	HOMO-1→LUMO (88)<br>HOMO-1→LUMO+1 (2)<br>HOMO→LUMO (4)<br>HOMO→LUMO+1 (6)	367		0.0729
3A	HOMO-1→LUMO (8)<br>HOMO-1→LUMO+1 (23)<br>HOMO-1→LUMO+2 (3)<br>HOMO→LUMO+1 (66)	365		0.7421
4A	HOMO-1→LUMO+1 (74)<br>HOMO→LUMO+1 (23)<br>HOMO→LUMO+2 (3)	340		0.3936
6A	HOMO-2→LUMO (100)	293		0.0821
8A	HOMO-6→LUMO+1 (22)<br>HOMO-5→LUMO+1 (43)<br>HOMO-3→LUMO+1 (13)<br>HOMO-2→LUMO (5)<br>HOMO→LUMO+2 (17)	264		0.0658

[a] bands with f>0.05 presented

Table S8. Shapes of molecular orbitals participating in electronic transitions in **1-**Ga^{3+}

Orbital	Energy, Ev	View
HOMO-6	-11.34	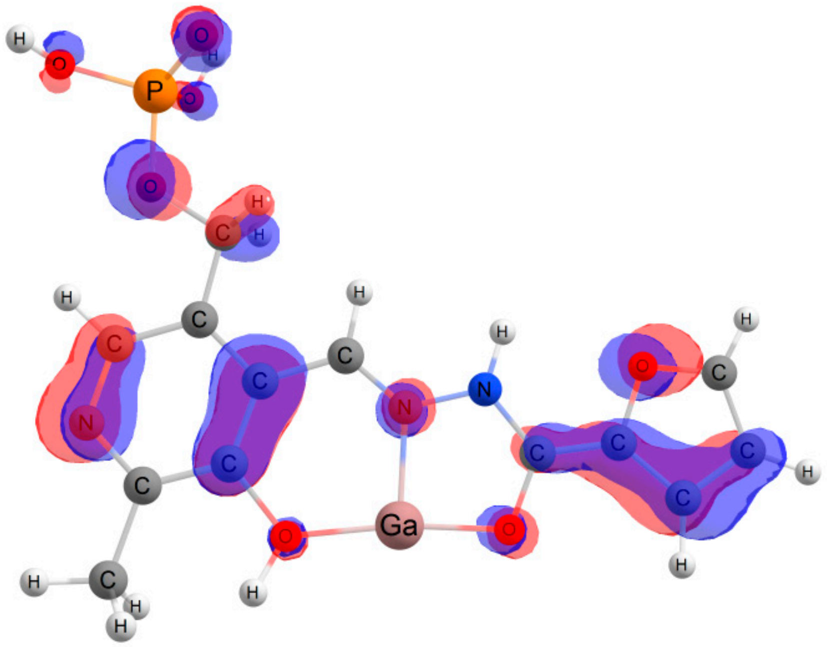
HOMO-5	-11.09	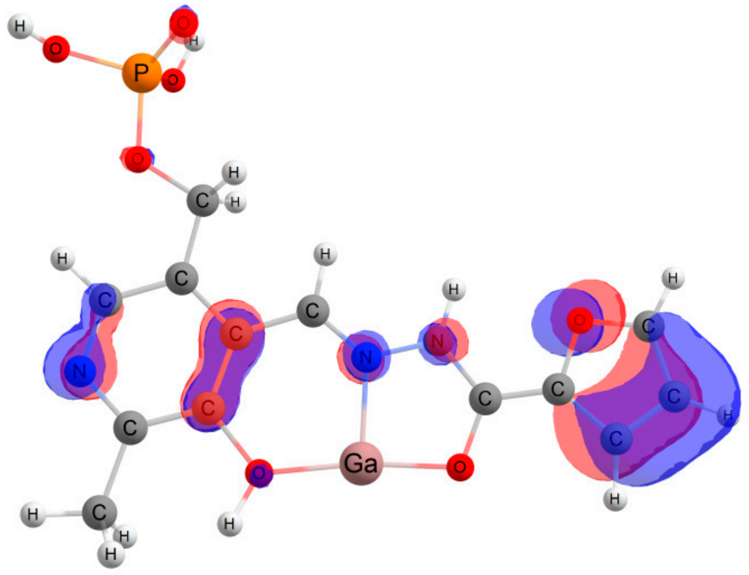

HOMO-4	-10.68	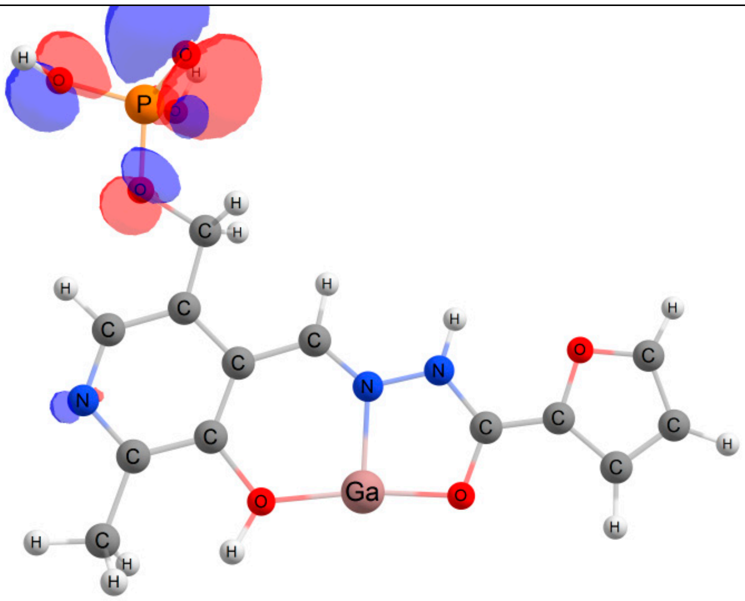
HOMO-3	-10.62	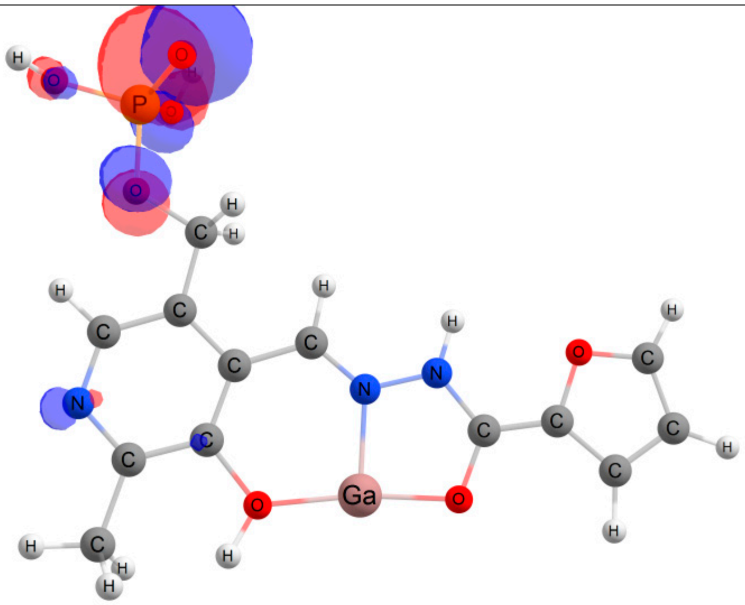
HOMO-2	-10.44	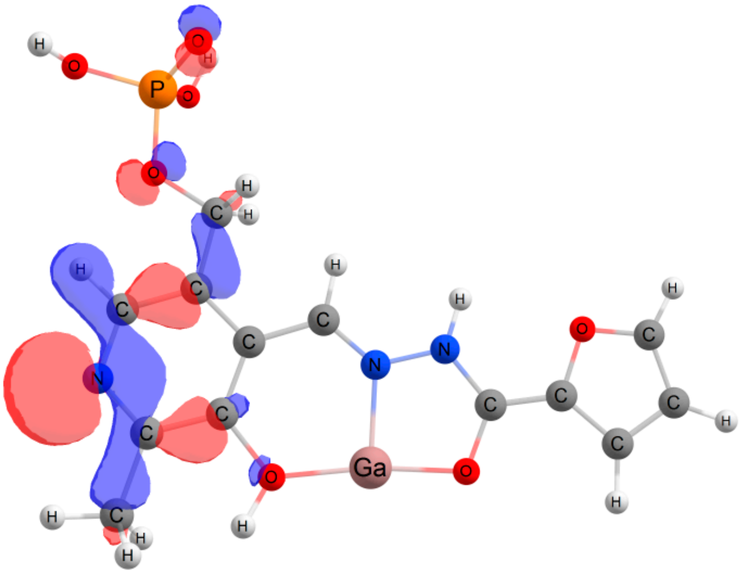

HOMO-1	-10.05	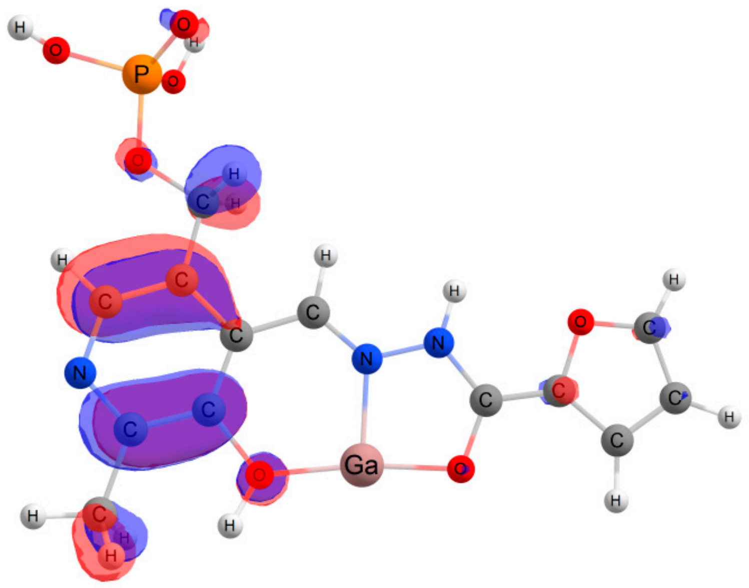
HOMO	-9.64	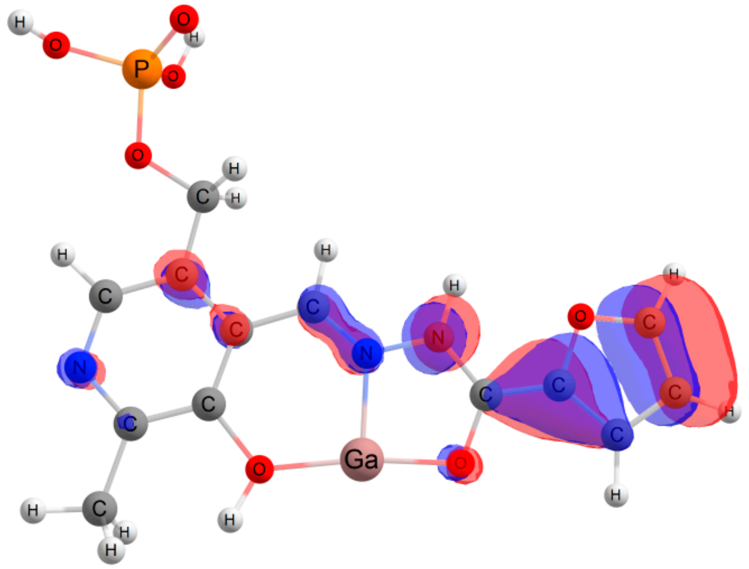
LUMO	-4.33	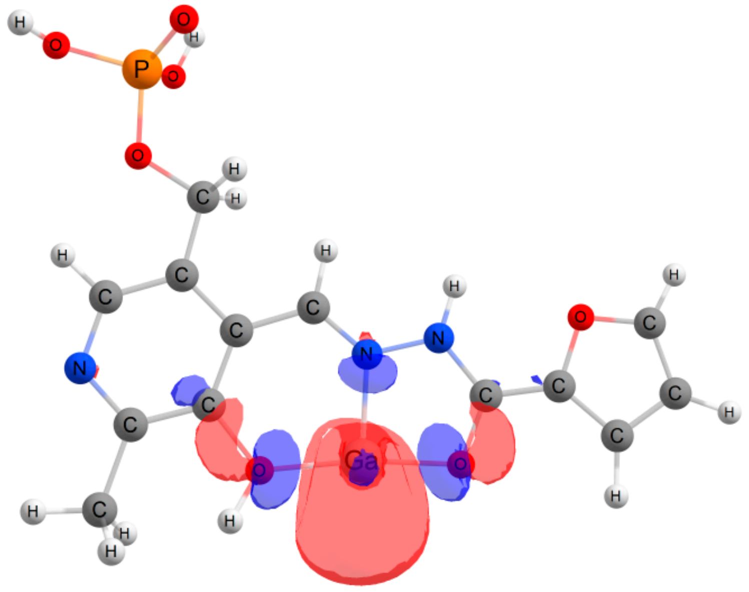

LUMO+1	-3.95	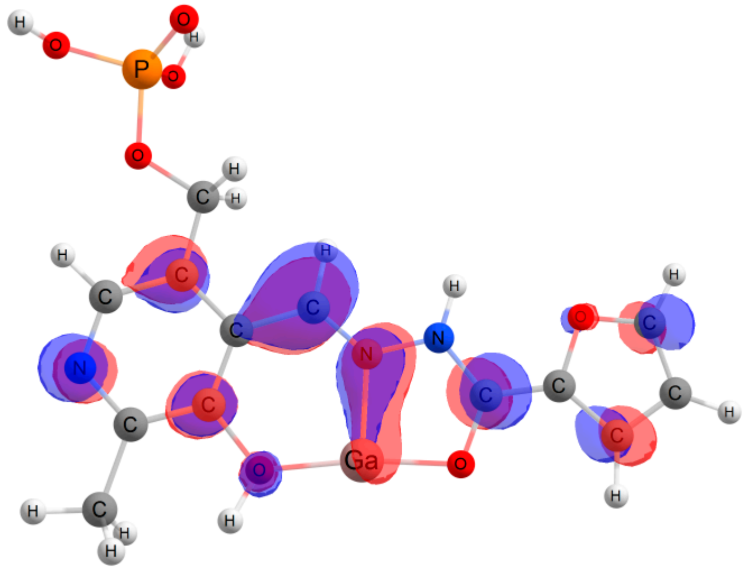
LUMO+2	-2.32	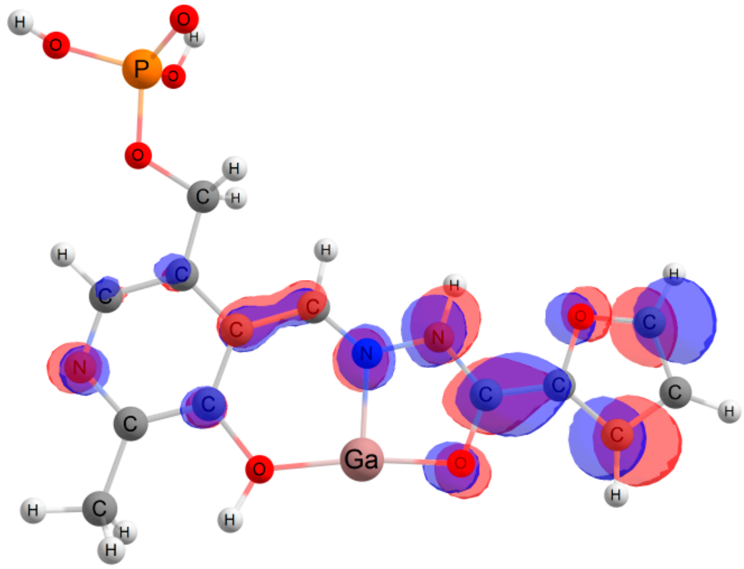

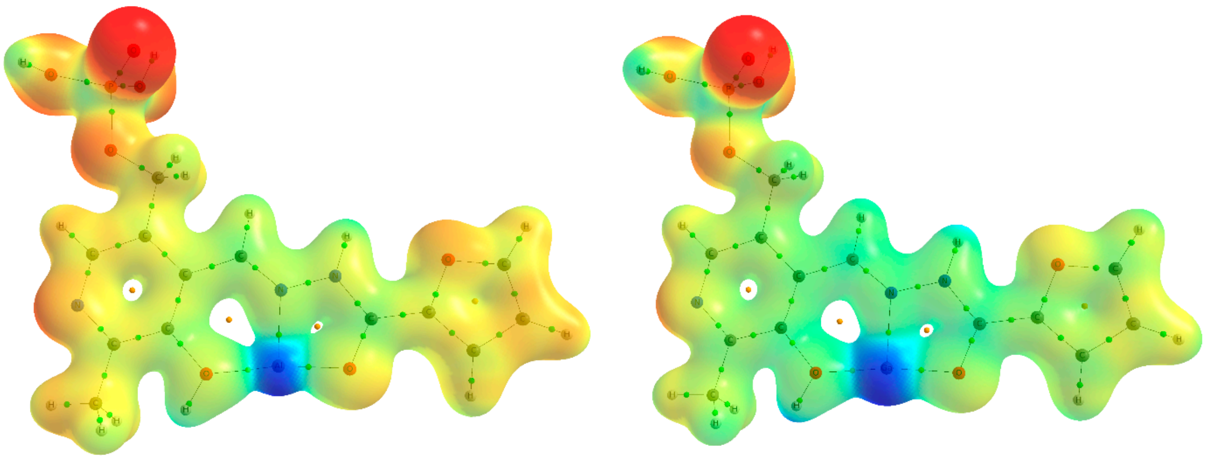

(a) (b)

Figure S12. The molecular electrostatic potential (MEP) mapped on the isodensity surface (0.04 a.u.) for **1**+Al^{3+} (a) and **1**+Ga^{3+} (b)
